# The immune cell landscape of peripheral blood mononuclear cells from PNS patients

**DOI:** 10.1038/s41598-021-92573-6

**Published:** 2021-06-22

**Authors:** Qing Ye, Chao Zhou, Sisi Li, Jingjing Wang, Fei Liu, Zhixia Liu, Jianhua Mao, Haidong Fu

**Affiliations:** 1 Department of clinical laboratory, The Children’s Hospital of Zhejiang University School of Medicine, National Clinical Research Center for Child Health, 3333 Binsheng Road, Hangzhou, 310003 Zhejiang China; 2 Zhejiang University City College, Hangzhou, China; 3 Department of Nephrology, The Children’s Hospital, Zhejiang University School of Medicine, National Clinical Research Center for Child Health, 3333 Binsheng Road, Hangzhou, China

**Keywords:** Kidney, Kidney diseases

## Abstract

Existing research suggests that the human immune system and immune cells are involved in the pathogenesis of nephrotic syndrome, but there is still a lack of direct evidence. This study tried to analyze the profiling of immune cells in the peripheral blood of steroid-sensitive nephrotic syndrome (SSNS) patients and steroid-resistant nephrotic syndrome (SRNS) patients before and after standard steroid treatment to clarify the immunological mechanism of nephrotic syndrome patients. The number and proportion of CD4 + T cells in patients with nephrotic syndrome remained unchanged. However, there is an imbalance of Th1 and Th2 and an excessive increase of Th17 cells. The number of CD8 + T cells and the number of effector CD8 + T cells in them increased significantly, but only in SSNS, the number of activated CD8 + T cells increased, and the number of activated T_reg_ cells decreased significantly. Nephrotic syndrome patients also have B cell disorder, and it is more prominent in SSNS patients. Compared with the normal control, only the number of B cells and plasmablast in SSNS patients increased significantly (Z = − 2.20, *P* = 0.028). This study also observed that transitional B cells decreased in both SSNS and SRNS patients, but SSNS patients' decrease was lower than in SRNS patients. Compared with normal controls, monocytes in patients with nephrotic syndrome decreased significantly. The main reason was that Non-classical Monocyte decreased, while Classical Monocyte increased slightly. The total number of NK cells did not change, but the internal cell subgroups' composition occurred. Changes, realized as CD56hi NK cells increased, CD56low NK cells decreased; and the above trend is more evident in SSNS patients. Patients with nephrotic syndrome have immune disorders, including T cells, B cells, Monocytes, and NK cells. It can be confirmed that immune factors are involved in the pathogenesis of the nephrotic syndrome.

## Introduction

Nephrotic syndrome (NS) is a type of syndrome caused by the increased permeability of the glomerular filtration membrane, which increases the filtration of plasma proteins and causes a large amount of proteinuria, and causes a series of characteristic pathologies—changing clinical syndrome. Such patients are often clinically manifested with large proteinuria, hypoalbuminemia, edema, hyperlipidemia, etc.^1^. Depending on nationality and race, the annual incidence of NS is 1.15–16.9/10,0000^1,2^. Moreover, about 10% to 20% of the children are resistant to glucocorticoid therapy, called steroid-resistant nephrotic syndrome (SRNS)^3^. The probability that children with SRNS will progress to end-stage renal disease five years after diagnosis is 8–35%, and the probability of progressing to end-stage renal disease 15 years after diagnosis is 24–66%. As a result, the disease has brought a heavy burden to society and families. The etiology of childhood nephrotic syndrome includes immune factors, genetic factors, and secondary causes (including infection, tumor, toxin, protein degeneration, allergies, chemical substances, etc.). Among them, immune factors are the most common cause, accounting for about 95% of all causes. The specific pathogenesis is believed to be related to the immune imbalance of the body. T cell dysfunction has always been considered as one of the important mechanisms of NS pathogenesis. This theory is mainly based on the following evidence: the measles virus can significantly inhibit the body's cellular immune function, and some patients with NS have been relieved of their original nephrotic syndrome symptoms after being infected with the measles virus^4^. Drugs that selectively inhibit T lymphocytes' function, such as cyclosporine/tacrolimus, have been helpful for some NS; T lymphocytes isolated from NS patients are cultured in vitro the isolated culture supernatant is injected. After administrating these supernatants to rats, it will induce a large amount of proteinuria^5^. The onset of thymoma patients and some patients with Hodgkin's disease are related to abnormal T lymphocyte function. It is clinically observed that this patient is prone to nephrotic syndrome. The above studies have shown that the onset of NS is closely related to the number and function of T lymphocytes.

In recent years, clinical studies have found that when patients with steroid-sensitive nephrotic syndrome (SSNS) relapse, the number of B cells in the peripheral blood increases significantly, and among steroid-dependent nephrotic syndrome (SDNS), there are a large number of activated B cells in the body. In contrast, the number of B cells in patients with SSNS in remission has decreased significantly^6^. The results of multiple multicenter clinical studies worldwide have shown that rituximab can be successfully used in the treatment of NS, especially in the treatment of refractory nephrotic syndrome^7^. Glucocorticoids have been shown to be unable to effectively inhibit B cells' activation in SDNS or SRNS patients while eliminating B cells using cyclophosphamide and rituximab can induce long-term remission in patients with SDNS after drug withdrawal. In addition, the number of memory B cells, especially switching memory B cells, can predict recurrence after rituximab treatment^8^. These findings indicate that abnormal B cell function plays a more important role in NS.

In summary, we can be sure that the human immune system and immune cells are involved in nephrotic syndrome's pathogenesis. However, the current findings are based on some side evidence. So far, there is no direct evidence on the status of immune cells in patients with nephrotic syndrome. Therefore, this article attempts to analyze the profiling of immune cells in the peripheral blood of SSNS patients and SRNS patients before and after standard steroid treatment to clarify the immunological mechanism of nephrotic syndrome patients.

## Result

### Demographic and clinical characteristics of patients with SRNS or SSNS before and after treatment

In this study, 26 patients with SRNS and 59 patients with SSNS were included. Thirty healthy people served as normal controls. Among SRNS patients, 18 cases were male, accounting for 69.2%; median age was 5.9 years; the pathological types were minimal change disease (MCD) 2 cases, mesangial proliferative glomerulonephritis (MsPGN) 5 cases, focal segmental glomerulosclerosis (FSGS) 5 cases, endocapillary proliferative glomerulonephritis (EPGN) 2 cases, and the remaining 2 cases had no renal biopsy. Among SSNS patients, 45 were male, accounting for 76.3%; the mean age was 5.8 years; the pathological types were MCD 13 cases, MsPGN 1 case, EPGN 1 case, and the remaining 44 cases had no renal biopsy. All patients underwent peripheral blood immunophenotyping before admission and after a standard hormonal course. At the same time, physical examination and related laboratory tests were performed. See Table 1 for specific data.

**Table 1:** Demographic and clinical characteristics of patients with SRNS or SSNS before and after treatment.

		SRNS BT		SRNS AT	SSNS BT		SSNS AT
Sex, male, n (%)			18 (69.2)			45 (76.3)	
Age, year			5.9 ± 4.3			5.8 ± 4.4	
	Median (min, max)		3.6 (1.2, 12.0)			4.9 (1.0, 13.0)	
	(95% CI)		3.8 to 7.9			2.6 to 8.9	
Weight, kg		22.9 ± 7.7		23.5 ± 7.5	22.2 ± 15.6		21.3 ± 8.3
	Median (min, max)	26.4 (13.0, 29.9)		27.1 (13.3, 31.5)	16.6 (10.0, 71.2)		18.4 (11.3, 75.7)
	(95% CI)	14.8 to 31.0		15.9 to 33.4	17.7 to 26.7		18.9 to 28.9
BP, mmHg	Systolic	111.5 ± 10.7		108.9 ± 11.4	103.7 ± 18.5		109.4 ± 18.0
	Median (min, max)	112 (98, 125)		110 (88, 129)	105 (9, 132)		108 (79, 150)
	(95% CI)	100.2 to 122.8		103.4 to 114.4	98.3 to 109.0		96.5 to 122.3
	Diastolic	70.8 ± 9.8		65.7 ± 12.0	63.4 ± 16.4		67.2 ± 17.9
	Median (min, max)	71 (59, 84)		64 (47, 84)	65 (4, 91)		66 (48, 112)
	(95% CI)	60.5 to 81.2		59.9 to 71.5	58.7 to 68.1		54.4 to 80.0
Serum albumin, g/L		17.6 ± 2.7		20.4 ± 8.9	18.0 ± 8.2		24.6 ± 5.7
	Median (min, max)	18.0 (12.7, 20.7)		19.4 (3.2, 37.7)	16.0 (10.7, 61.1)		24.2 (17.0, 32.7)
	(95% CI)	14.8 to 20.4		16.1 to 24.7	15.7 to 20.4		20.5 to 28.7
Creatinine, μmol/L		2908.5 ± 1546.8		3937.7 ± 3342.7	4274.5 ± 3636.1		6225.7 ± 5449.7
	Median (min, max)	2838 (679, 4655)		3330 (124, 12809)	2756 (103, 17479)		5718 (223, 15552)
	(95% CI)	1285.3 to 4531.7		2326.6 to 5548.9	3230.1 to 5318.9		2327.2 to 10124.2
24-hour urinary protein mg/24h		1463.8 ± 1036.1		1086.0 ± 411.3	1053.3 ± 670.6		1013.5 ± 920.2
	Median (min, max)	1300 (400, 5073)		1125 (300, 1750)	855 (380, 2000)		700 (250, 5800)
	(95% CI)	964.5 to 1963.2		791.8 to 1380.2	349.6 to 1757.1		749.2 to 1277.8
Urinary protein/urinary creatinine		4.1 ± 2.7		3.7 ± 2.7	5.3 ± 3.9		2.5 ± 1.9
	Median (min, max)	5.1 (0.2, 7.3)		3.7 (0.1, 11.2)	3.7 (0.2, 15.0)		1.9 (0.3, 6.7)
	(95% CI)	1.2 to 7.0		2.4 to 5.0	4.2 to 6.4		1.2 to 3.9
Blood glucose, mmol/L		6.2 ± 0.7		6.5 ± 1.6	6.1 ± 2.7		6.0 ± 1.3
	Median (min, max)	6.1 (5.3, 7.2)		6.3 (4.8, 11.6)	5.6 (2.3, 23.0)		5.7 (4.6, 8.8)
	(95% CI)	5.4 to 6.9		5.8 to 7.3	5.3 to 6.9		5.1 to 6.9
Blood triglyceride, mmol/L		2.3 ± 1.8		2.9 ± 1.7	2.6 ± 1.4		2.4 ± 2.1
	Median (min, max)	2.2 (0.8, 6.8)		1.4 (0.8, 5.2)	2.3 (1.2, 7.7)		1.7 (0.9, 8.0)
	(95% CI)	2.0 to 3.7		0.5 to 4.2	2.2 to 3.1		0.9 to 4.0
Blood cholesterol, mmol/L		7.4 ± 2.9		9.6 ± 3.5	10.3 ± 3.2		7.9 ± 2.9
	Median (min, max)	9.2 (5.2, 13.6)		7.4 (3.5, 13.6)	10.3 (4.8, 17.8)		7.7 (3.4, 12.8)
	(95% CI)	5.9 to 13.3		6.0 to 8.8	9.4 to 11.3		5.8 to 9.9
**Histopathologic diagnosis, n (%)**							
	MCD		12 (46.2)			13 (22.0)	
	MsPGN		5 (19.2)			1 (1.7)	
	EPGN		2 (7.7)			1 (1.7)	
	FSGS		5 (19.2)			0 (0.0)	
	No biopsy		2 (7.7)			44 (74.6)	

### Changes of T cells in peripheral blood of patients with nephrotic syndrome

Compared with normal controls, the T cell count of patients with nephrotic syndrome has a significant increase (Z = − 2.09, *P* = 0.037), and there is no difference in the proportion and absolute value of CD4 + T cells. However, there are differences in their cell types. The specific manifestation is that the number of Th1 cells (Z = − 3.04, *P* = 0.002) and the proportion (Z = − 4.57, *P* < 0.001) are significantly reduced; the proportion of Th2 cells increases (Z = − 3.76, *P* < 0.001); The count of Th17 cells was significantly increased (Z = − 2.15, *P* = 0.031); and the above data was between SSNS and SRNS, and there was no significant change before and after their respective treatments. In T_reg_ cells (CCR4 + CD25 + CD127low), the proportion of CD45RO + memory T_reg_ cell in SRNS patients was significantly reduced (Z = − 3.62, *P* = 0.002), and it can quickly recover after treatment; There was no change about CD45RO- naive T_reg_ cell, but HLA-DR + activated T_reg_ cells decreased significantly in SSNS patients (*P* < 0.05). In CD4 + T cells, naive CD4 + T cell (CCR7 + CD45RA +) significantly increased, central memory CD4 + T cell (CCR7 + CD45RA−) and effector memory CD4 + T cell (CCR7− CD45RA−) decreased, and effector CD4 + T The cell (CCR7-CD45RA +) and activated CD4 + T cells (CD38 + HLA-DR +) did not change. Except that the percentage of naive CD4 + T cells in SRNS will decrease significantly after treatment, other indicators have not changed significantly between SSNS and SRNS, and before and after their respective treatments.

**Figure 1:**
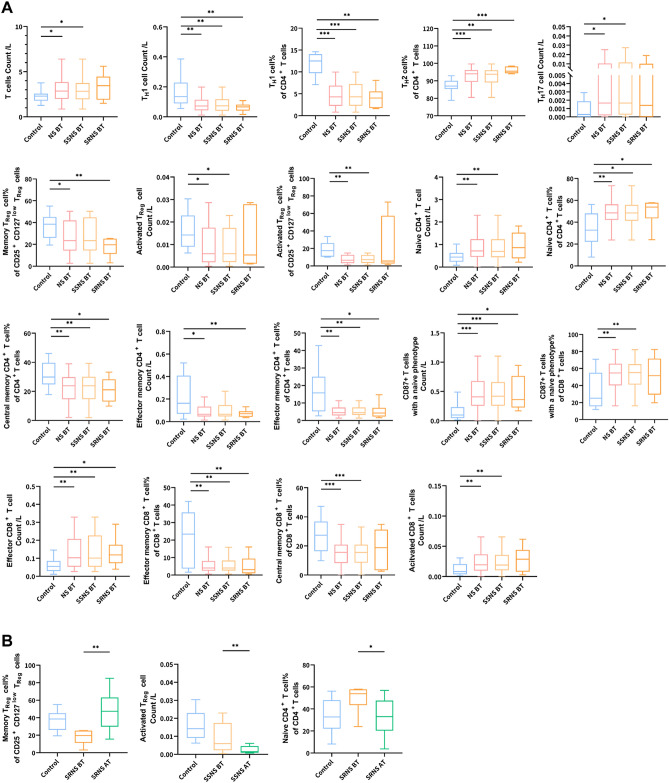
Changes of T cells in peripheral blood of patients with nephrotic syndrome. **(A)** Changes of T cells in peripheral blood of patients with nephrotic syndrome before treatment compared with normal control; **(B)** changes of T cells in peripheral blood of patients with nephrotic syndrome before and after treatment. *Control* normal control (n = 30), *NS* nephrotic syndrome (n = 85), *SSNS* steroid-sensitive nephrotic syndrome (n = 59), *SRNS* steroid-resistant nephrotic syndrome (n = 26), *BT* before treatment, *AT* after treatment.

Compared with normal controls, the number of CD8 + T cells in patients with nephrotic syndrome and the number of CD8 + T cells with a naive phenotype (CCR7 + CD45RA +) and effector CD8 + T cells (CCR7-CD45RA +) were significantly increased (*P* < 0.05). The percentage of effector memory CD8 + T cell (CCR7− CD45RA−) decreased significantly. The proportion of Central memory CD8 + T cells (CCR7 + CD45RA−) in SSNS decreased significantly, while the number of activated CD8 + T cells (CD38 + HLA-DR +) increased; there was no corresponding change in SRNS. See Supplemental Table 2–3 and Fig. 1 for specific data.

To sum up, although the number and proportion of CD4 + T cells in patients with nephrotic syndrome have not changed. However, there is an imbalance of Th1 and Th2 and an excessive increase of Th17 cells. The number of CD8 + T cells and the number of Effector CD8 + T cells in them both increased significantly, but only the number of Activated CD8 + T cells in SSNS increased, and the number of HLA-DR + Activated T Reg cells decreased significantly.

### Changes of B cells in peripheral blood of patients with nephrotic syndrome

**Figure 2:**
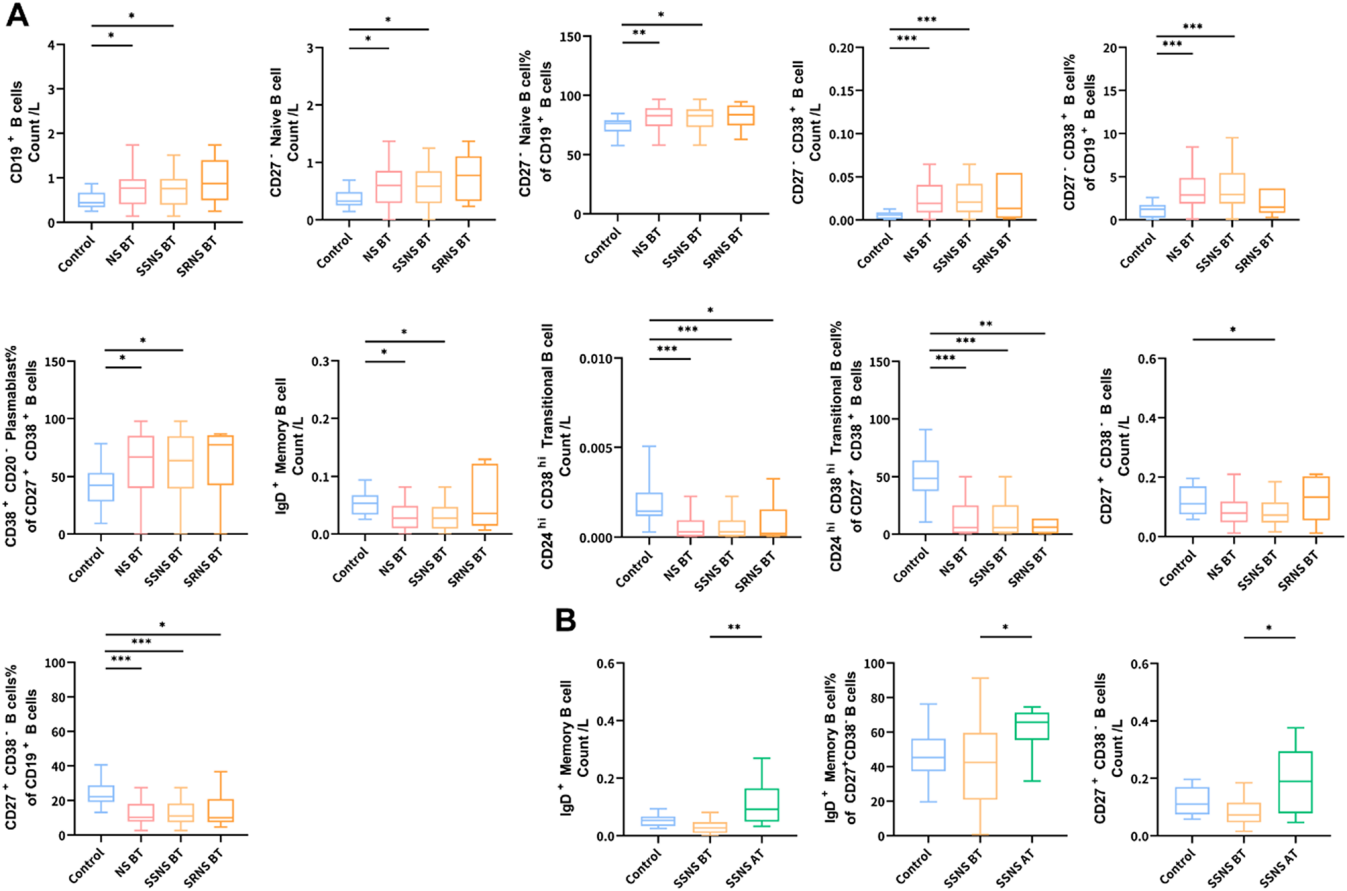
Changes of B cells in peripheral blood of patients with nephrotic syndrome. **(A)** Changes of B cells in peripheral blood of patients with nephrotic syndrome before treatment compared with normal control; **(B)** changes of B cells in peripheral blood of patients with nephrotic syndrome before and after treatment. *Control* normal control (n = 30), *NS* nephrotic syndrome (n = 85), *SSNS* steroid-sensitive nephrotic syndrome (n = 59), *SRNS* steroid-resistant nephrotic syndrome (n = 26), *BT* before treatment, *AT* after treatment.

Compared with normal controls, only SSNS patients had a significant increase in B cells (Z = − 2.20, *P* = 0.028). Among them, only the number and proportion of CD27-Naive B cells and CD27-CD38 + B cells in SSNS patients increased, and the percentage of Plasmablast (CD20-CD38 +) in CD27 + CD38 + B cells increased, and the number of IgD + Memory B cells decreased, and IgD + Memory B cell will recover after treatment. In addition, Transitional B cells (CD24hi CD38hi) and CD27 + CD38− B cells were reduced in SSNS patients and SRNS patients, but the decline in SSNS patients was even lower. Moreover, only the number of CD27 + CD38– B cells in SSNS patients will increase significantly after treatment (Z = − 2.59, *P* = 0.010). It can be seen that the disorder of B cells is more prominent in SSNS patients. See Supplemental Tables 2–3 and Fig. 2 for specific data.

### Changes of monocytes and DC cells in peripheral blood of patients with nephrotic syndrome

**Figure 3:**
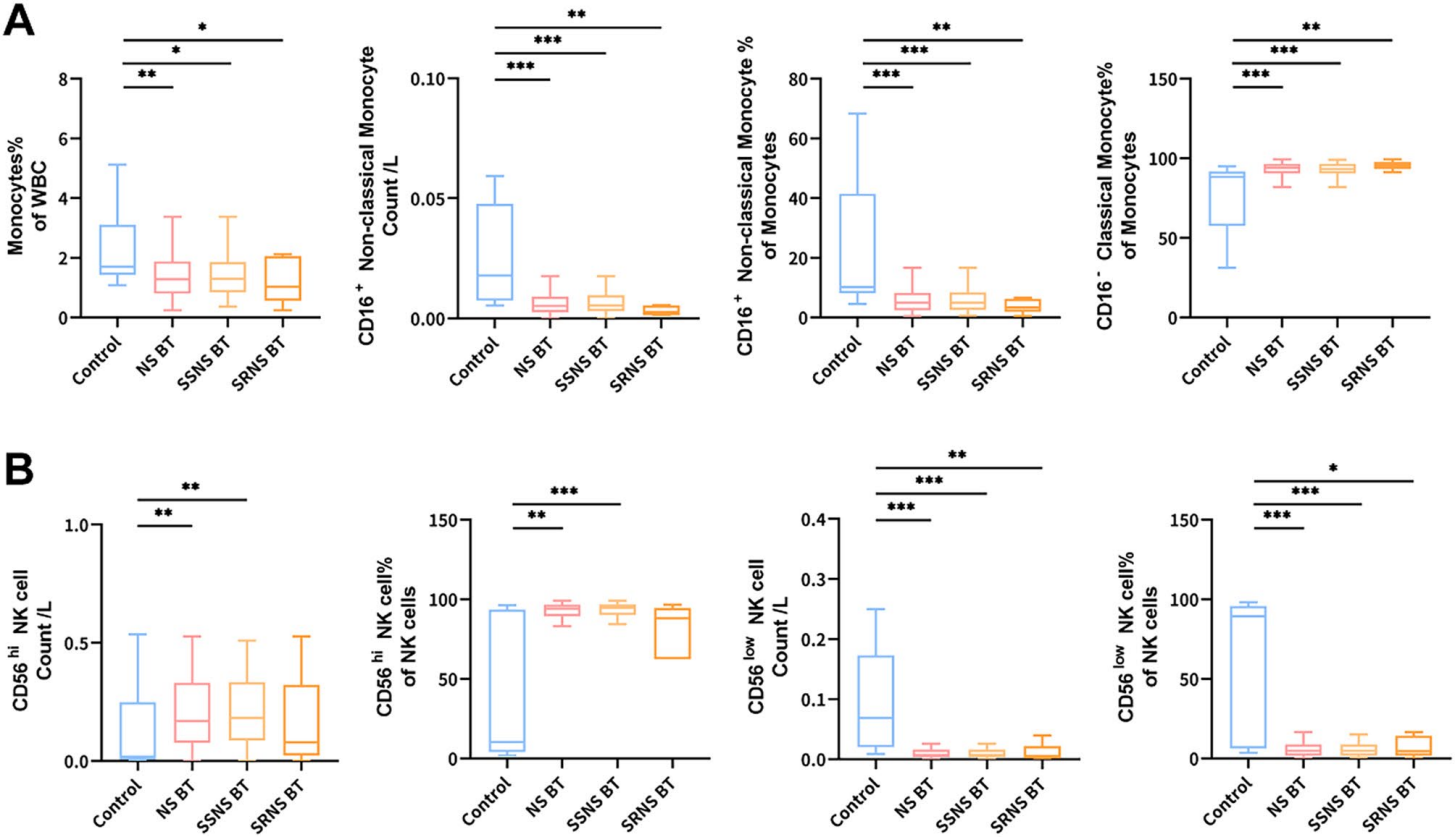
Changes of Monocytes and NK cells in peripheral blood of patients with nephrotic syndrome. **(A)** Changes of Monocytes in peripheral blood of patients with nephrotic syndrome before treatment compared with normal control; **(B)** changes of NK cells in peripheral blood of patients with nephrotic syndrome before treatment compared with normal control. *Control* normal control (n = 30), *NS *nephrotic syndrome (n = 85), *SSNS* steroid-sensitive nephrotic syndrome (n = 59), *SRNS* steroid-resistant nephrotic syndrome (n = 26), *BT* before treatment.

Compared with normal controls, Monocytes in patients with nephrotic syndrome decreased significantly. The main reason is that Non-classical Monocyte (CD16 +) has decreased, while Classical Monocyte (CD16-) has increased slightly. Furthermore, these changes are more evident in SSNS patients than in SRNS patients. The DC cells did not change significantly. See Supplemental Tables 2–3 and Fig. 3 for specific data.

### Changes of NK cells in peripheral blood of patients with nephrotic syndrome

There is no change in NK cells in patients with nephrotic syndrome, but the composition of its internal cell subgroups has changed. It turns out that CD56hi NK cells increase and CD56low NK cells decrease. This trend is more evident in SSNS patients than SRNS patients. See Supplemental Tables 2–3 and Fig. 3 for specific data.

## Discussion

Sensitivity to steroids and immunosuppressive therapy is the most important clinical argument supporting some nephrotic syndrome's immune origin. In addition, the study also observed spontaneous nephrotic syndrome remission after measles infection^9^; Nephrotic syndrome in Hodgkin's lymphoma and other T-cell lymphomas^10^ are regressed after chemotherapy; allergic reactions to various allergens and poisons in the body will cause the progression of nephrotic syndrome^11^. The above findings further suggest that the pathogenesis of the nephrotic syndrome is related to the dysfunction or abnormal regulation of T lymphocytes^12^. By detecting the immune cells in the peripheral blood of patients with nephrotic syndrome, this study found that patients with nephrotic syndrome have an imbalance of Th1 and Th2; the excessive increase of Th17 cells; the number of CD8 + T cells, and the number of CD8 + T cells with a naive phenotype and effector CD8 + T cells were also significant Elevated. As we know, in contrast to CD4 + T cells, CD8 + T cells can revert their expression of the activated/memory marker CD45RO to the naive marker CD45RA^13^. Besides, there is an increase in the number of activated CD8 + T cells and a significant decrease in HLA-DR + Activated Treg cells in SSNS. The level of HLA-DR + Activated T_reg_ cell can be quickly restored after effective treatment. Other studies also suggest an imbalance of Th1/Th2^14^ and Th17/T_reg_ dysregulation^15^. The study also found that levamisole can play a therapeutic role by up-regulating Th1 up-regulation^16^, and MMF reduces Th17^17^ and increases T_reg_^18^.

Additionally, Th2^19^ and Th17^20^ up-regulation and down-regulation of T_reg_ cells^20^ are closely related to the recurrence of the disease. Studies on genetic polymorphisms in the variable region of the TCR β chain have shown that there is selective recruitment of some V region β gene families in the peripheral blood CD8^+^ T cells of patients with frequent relapse of kidney disease^21^. These results also suggest that CD8^+^ T cells can undergo clonal expansion during the ongoing disease activity.

In recent years, clinical studies have found that the number and activation level of B cells in patients' peripheral blood with nephrotic syndrome are related to the disease outcome^6^. Moreover, multiple multicenter clinical research results worldwide show that rituximab can be successfully used in nephrotic syndrome's treatment, and the effect is significant^22^. At that, B cells also play an important role in the pathogenesis of the nephrotic syndrome. Our research also found that B cell disorders exist in patients with nephrotic syndrome, and it is more prominent in patients with SSNS. Compared with the normal control, only the number of B cells and plasmablast in SSNS patients increased significantly (Z = − 2.20, *P* = 0.028). B cell depletion strategies have enabled dissection of B cells' functional role in the pathogenesis of various nephrotic states. In membranous glomerulonephritis, rituximab therapy is effective in two-thirds of patients and is presumed to act through a reduction in the levels of pathogenic anti -phospholipase A2 receptor or anti-thrombospondin type 1 domain-containing protein 7A antibodies^23^. Significant increases in T_reg_ cells in responders following rituximab therapy have also been noted^24^. So, rituximab might correct T_reg_ cell: Th17 cell and CD4 + T cell: CD8 + T cell ratio imbalances found in the active phase of disease by increasing T_reg_ cells' levels^20^. In addition, this study also observed that Transitional B cells decreased in both SSNS and SRNS patients, but the decrease in SSNS patients was even lower. During ontogeny, bone marrow-derived immature B-cell precursors migrate into the periphery as transitional B-cells (CD24^high^CD38^high^). Some of these are considered regulatory B (B_reg_) cells, which play an immunomodulatory role via the secretion of interleukin-10 (IL-10), IL-35, and transforming growth factor β (TGF-β)^25,26^. B_reg_ cells suppress immunopathology by prohibiting the expansion of pathogenic T cells and other pro-inflammatory lymphocytes^25,27^. Decreased numbers and/or function of B_reg_ cells have been described in lupus nephritis and ANCA-associated vasculitis, contributing to disease pathogenesis and/or relapse^28^. Furthermore, the transitional B-cell can regulate T-cell activation by inhibiting Th17 cell polarization^29^, promoting T_reg_-cell induction^30^, and reducing IFN-γ and TNF production by Th1 cells^25,31^ may be involved in the pathogenesis of SSNS.

Compared with normal controls, Monocytes in patients with nephrotic syndrome decreased significantly. The main reason is that CD16 + Non-classical Monocyte has declined, while CD16- Classical Monocyte has increased slightly. These changes are more evident in SSNS patients than in SRNS patients. Monocytes are bone marrow-derived leukocytes with functional capacities including phagocytose, antigen presentation, and cytokine production. In the late 1980s, Passlik et al. identified different monocyte subsets based on the expression of the surface antigen CD16^32^. Classical monocytes are critical components of innate immunity, represent the largest monocytes' population, and are essential scavenger cells. Non-classical monocytes have antagonizing functions to classical monocytes and promote neutrophil adhesion at the endothelial interface via the secretion of TNF-α^33^ and do not reach the classical monocyte production levels of pro-inflammatory cytokines^34^. Thus, it can be seen, the imbalance of classical monocyte and non-classical monocyte is also involved in the pathogenesis of the nephrotic syndrome.

There is no change in NK cells in patients with nephrotic syndrome, but the composition of its internal cell subgroups has changed. It turns out that CD56hi NK cells increase and CD56low NK cells decrease, and this trend is more evident in SSNS patients. NK cells fall into two categories based on the quantity of CD56 on their surface: CD56hi NK cell and CD56lo NK cell^35^. These two types of conventional NK cells have different effector functions. CD56lo NK cells are highly cytotoxic and are mostly present in peripheral blood, which constitutes about 90% of NK cells in the blood, whereas CD56hi NK cells, which constitute about 5–15% of NK cells, are mainly present in lymph nodes and perform regulatory functions via releasing some cytokines and responding to some others^36^. It can be seen that the imbalance of CD56hi NK cell and CD56low NK cell is also involved in the pathogenesis of the nephrotic syndrome.

In summary, patients with nephrotic syndrome have immune dysfunction, including T cells, B cells, Monocytes, and NK cells. Immune factors are involved in the pathogenesis of the nephrotic syndrome.

## Materials and methods

This study included first-onset patients who came to our hospital for nephrotic syndrome in 2020. All patients need to undergo genome whole-exome sequencing and other related laboratory tests before being included in this study to exclude patients caused by hereditary and secondary factors. The patients who were finally enrolled in this study collected 3–5 ml of venous blood into the EDTA anticoagulation tube before and after the standard steroid treatment and immediately sent it to the laboratory. The laboratory used BD Facscanto II Flow Cytometry for whole blood test immediately after receiving the sample and setting standard beads' fluorescence to defined target channels for reproducible setup across instruments. All antibodies and reagents were sourced from BD Pharmingen (Supplemental table 4). All detections are performed by a professional using automated gating algorithms and analyzed using Diva software. The immunophenotyping strategy refers to Maecker HT et al. in Nat Rev Immunol ^37^. See Supplemental Fig. 1–5 for Gating strategy. Simultaneously, healthy volunteers' peripheral blood was collected for analysis, and the results were used as normal controls. After all the specimens were tested, the patients were divided into SSNS and SRNS according to their sensitivity to steroid treatment. Analyze the differences between SSNS patients and SRNS patients and normal controls and the differences between them and before and after treatment. The remaining blood of healthy subjects who come to our hospital for physical examination was collected as normal control. All experiments were reviewed and approved by the Ethics Committee of the children’s Hospital, Zhejiang University School of Medicine (2019-IRB-139), and were performed according to their regulations and guidelines. All subjects gave written informed consent before they participated in this study.

### The operation method of peripheral blood immunophenotyping

1. Take five tubes of 4 ml flow tube and mark;
2. Add 5 μl of the fluorescently labeled monoclonal antibody according to the scheme below (Supplemental Table 1).
3. Add 50 μl of blood to each tube, vortex to mix, and react for 30 min at room temperature in the dark.
4. Add 1 ml of the hemolytic agent to each tube, vortex to mix, and react for 15 min in the dark.
5. Add 3 ml of PBS to each tube, 300*g*, and centrifuge for 5 min to wash.
6. BD FACSCanto™ II Flow cytometry is tested on the computer.
7. Diva software analysis refers to the protocol published by Maecker HT et al. in Nat Rev Immunol and uses cell surface CD molecules to set up gates and define analysis.

### Statistical analysis method

All variables were described as median (interquartile range). The Shapiro–Wilk test was used to determine whether the samples are normally distributed or not. Statistical significance was analyzed using independent samples t-test or Mann–Whitney U test for comparing two conditions, and one-way ANOVA with Bonferroni test or Kruskal–Wallis test with Dunn test for multiple comparisons. All statistical analyses were performed using SPSS 25.0 software (IBM Inc). *P* < 0.05 was considered statistically significant.

### Ethics approval

This research was conducted ethically in accordance with the World Medical Association Declaration of Helsinki. The study protocol was approved by our institute’s committee on human research.

### Consent for publication

All authors declare their consent for publication.

## Supplementary Information

Supplementary Table 1.

Supplementary Table 2.

Supplementary Table 3.

Supplementary Table 4.

Supplementary Figure 1.

Supplementary Figure 2.

Supplementary Figure 3.

Supplementary Figure 4.

Supplementary Figure 5.

Supplementary Legends.

## Data Availability

All data are available as requested.

## Code availability

Microsoft Office, IBM SPSS v21.
