# Supplementary material for: The immune cell landscape of peripheral blood mononuclear cells from PNS patients
Source: Sci Rep. 2021 Jun 22;11:13083. doi: 10.1038/s41598-021-92573-6 (PMC8219797; doi:10.1038/s41598-021-92573-6)
Supplement: Supplementary file 1 — Supplementary Table 1. [file 41598_2021_92573_MOESM1_ESM.pdf]

Supplemental table 1 Eight-color antibody panels proposed by the Human Immunophenotyping Consortium

Fluorochrome	Marker				
	T cells	T_{reg} cells	Th1, Th2, and Th17 cells	B cells	DCs, monocytes, and NK cells
PerCP	CD45	CD45	CD45	CD45	CD45
BV421	CD38	CD127	CD38	CD38	CD123
BV510	CD3	CD3	CD3	CD3	CD3, CD19, CD20
APC-R700	CD4	CD4	CD4	CD19	CD56
PE-CY7	CD8	CCR4	CD8	CD24	CD16
APC	CD45RA	CD45RO	CXCR3	CD20	CD14
BB515	CCR7	CD25	CCR6	IgD	CD11C
PE	HLA-DR	HLA-DR	HLA-DR	CD27	HLA-DR
