# Supplementary material for: The immune cell landscape of peripheral blood mononuclear cells from PNS patients
Source: Sci Rep. 2021 Jun 22;11:13083. doi: 10.1038/s41598-021-92573-6 (PMC8219797; doi:10.1038/s41598-021-92573-6)
Supplement: Supplementary file 2 — Supplementary Table 2. [file 41598_2021_92573_MOESM2_ESM.pdf]

Supplemental table 2 Differences in terms of cell subpopulations in HCs and untreated patients with SRNS or SSNS

Cell subpopulation	Unit	Control median (IQR)	NS median (IQR)	Z Value	t Value	P Value	SRNS median (IQR)	Z Value	t Value	P Value	SSNS median (IQR)	Z Value	t Value	P Value
**WBC**	Count /L	6.50 (5.78-7.95)	8.75 (6.34-11.18)	-2.84		0.004 **	9.94 (8.16-13.59)		2.71	0.038 *	8.70 (6.31-11.16)	-2.68		0.007 **
**T cells**	% of WBC	31.33 (28.26-41.46)	36.46 (26.78-43.91)		0.54	0.590	35.38 (26.13-40.93)		-0.12	0.908	36.46 (23.85-46.47)		0.80	0.430
	Count /L	2.29 (1.78-2.58)	2.85 (2.00-3.85)	-2.09		0.037 *	3.45 (1.90-4.46)		1.73	0.136	2.83 (1.94-3.74)	-2.00		0.045 *
**CD4$^+$ T cells**	% of T cells	58.20 (43.70-70.63)	57.40 (53.90-66.00)		0.24	0.813	50.20 (42.68-63.13)		-0.67	0.513	57.50 (54.05-66.20)		0.41	0.691
	Count /L	1.08 (0.87-1.55)	1.64 (1.05-2.39)	-1.87		0.062	1.50 (0.90-2.90)	-0.99		0.322	1.64 (1.07-2.37)	-1.87		0.062
**CXCR3$^+$ CCR6$^-$ T_H1 cell**	% of CD4$^+$ T cells	12.50 (9.72-14.05)	4.29 (2.26-6.87)	-4.57		<0.001 ***	4.02 (1.76-5.54)	-3.30		0.001 **	4.31 (2.28-7.20)	-4.40		<0.001 ***
	Count /L	0.14 (0.09-0.23)	0.07 (0.04-0.11)	-3.04		0.002 **	0.07 (0.04-0.08)	-2.64		0.008 **	0.07 (0.04-0.12)	-2.84		0.004 **
**CXCR3$^-$ CCR6$^-$ T_H2 cell**	% of CD4$^+$ T cells	87.00 (85.40-90.00)	94.00 (89.40-96.20)	-3.76		<0.001 ***	95.55 (94.38-98.28)		4.88	<0.001 ***	93.80 (89.10-96.00)	-3.47		0.001 **
	Count /L	0.95 (0.77-1.39)	1.57 (0.89-2.25)	-1.92		0.054	1.42 (0.88-2.82)	-1.40		0.161	1.57 (0.86-2.22)	-1.85		0.064
**CXCR3$^-$ CCR6$^+$ TH_{17} cell**	% of CD4$^+$ T cells	0.03 (0.00-0.20)	0.14 (0.02-0.61)	-1.78		0.075	0.11 (0.00-0.45)	-0.38		0.704	0.14 (0.02-0.64)	-1.90		0.058
	Count /L	0.0003 (0.0000-0.0019)	0.0017 (0.0002-0.0104)	-2.15		0.031 *	0.0014 (0.0000-0.0100)	-0.51		0.613	0.0017 (0.0003-0.0115)	-2.28		0.022 *
**CD25$^+$ CD127^{low} T_{Reg} cells**	% of CD4$^+$ T cells	6.46 (5.03-8.23)	5.90 (4.01-7.32)	-1.43		0.152	4.10 (2.28-6.39)	-2.06		0.039 *	6.21 (4.10-7.41)	-1.17		0.240
	Count /L	0.08 (0.06-0.13)	0.08 (0.05-0.15)	-0.10		0.917	0.05 (0.04-0.14)	-1.48		0.138	0.08 (0.05-0.15)	-0.41		0.679
**CD45RO$^+$ Memory T_{Reg} cell**	% of CD25$^+$ CD127^{low} T_{Reg} cells	38.50 (26.03-45.15)	23.50 (14.32-42.00)	-2.05		0.040 *	19.71 (11.19-25.18)		-3.62	0.002 **	23.50 (14.36-44.70)	-1.72		0.086
	Count /L	0.03 (0.02-0.05)	0.02 (0.01-0.04)	-1.00		0.317	0.02 (0.01-0.03)		-2.06	0.054	0.02 (0.01-0.05)	-0.71		0.477
**CD45RO$^-$ Naive T_{Reg} cell**	% of CD25$^+$ CD127^{low} T_{Reg} cells	61.25 (54.73-73.83)	55.90 (34.30-71.00)	-1.33		0.184	41.05 (29.70-76.03)		-1.39	0.213	57.90 (41.15-70.85)	-1.21		0.227
	Count /L	0.04 (0.03-0.09)	0.04 (0.02-0.08)	-0.76		0.447	0.02 (0.01-0.11)	-1.40		0.161	0.05 (0.02-0.08)	-0.56		0.574
**HLA-DR$^+$ Activated T_{Reg} cell**	% of CD25$^+$ CD127^{low} T_{Reg} cells	17.50 (10.65-26.10)	6.62 (3.29-12.70)	-2.78		0.005 **	5.72 (2.83-57.33)	-1.16		0.248	7.27 (3.42-12.60)	-2.85		0.004 **
	Count /L	0.014 (0.009-0.023)	0.006 (0.002-0.018)	-2.30		0.022 *	0.005 (0.002-0.028)	-1.24		0.216	0.006 (0.002-0.017)	-2.30		0.022 *
**Activated CD4$^+$ T cells**	% of CD4$^+$T cells	1.45 (0.57-2.07)	1.23 (0.82-2.29)	-0.37		0.709	1.10 (0.48-2.16)		0.01	0.991	1.31 (0.83-2.32)	-0.46		0.643
	Count /L	0.02 (0.00-0.03)	0.02 (0.01-0.04)	-1.36		0.175	0.02 (0.01-0.04)		0.36	0.722	0.02 (0.01-0.04)	-1.41		0.160
**Central memory CD4$^+$ T cell**	% of CD4$^+$T cells	29.85 (24.80-39.63)	23.80 (14.60-29.70)		-3.00	0.004 **	21.05 (13.05-28.28)		-2.37	0.029 *	23.80 (14.70-30.05)		-2.87	0.006 **
	Count /L	0.43 (0.22-0.56)	0.36 (0.26-0.47)	-0.60		0.551	0.37 (0.25-0.45)		-0.65	0.521	0.36 (0.25-0.47)		-0.66	0.513
**Effector CD4$^+$ T cell**	% of CD4$^+$T cells	11.75 (8.72-19.23)	11.30 (7.26-21.30)	-0.27		0.788	9.79 (8.24-14.50)	-0.49		0.621	12.00 (7.11-21.45)	-0.20		0.843
	Count /L	0.15 (0.09-0.29)	0.19 (0.09-0.36)	-0.97		0.332	0.19 (0.11-0.24)	-0.49		0.621	0.19 (0.09-0.52)	-0.98		0.329
**Effector memory CD4$^+$ T cell**	% of CD4$^+$T cells	15.80 (5.27-25.03)	4.50 (2.99-7.40)	-3.07		0.002 **	4.26 (2.28-7.28)	-2.23		0.026 *	4.50 (3.19-7.43)	-2.95		0.003 **
	Count /L	0.16 (0.07-0.41)	0.07 (0.05-0.13)	-2.01		0.044 *	0.07 (0.04-0.09)		-3.06	0.008 **	0.07 (0.05-0.15)	-1.90		0.057
**Naive CD4$^+$ T cell**	% of CD4$^+$T cells	32.70 (22.15-47.95)	48.50 (42.10-56.30)	-2.69		0.007 **	53.85 (43.50-57.75)	-2.14		0.032 *	48.40 (41.30-55.80)	-2.55		0.011 *
	Count /L	0.45 (0.24-0.63)	0.71 (0.45-1.24)	-2.70		0.007 **	0.86 (0.39-1.53)		1.91	0.106	0.70 (0.44-1.24)	-2.63		0.009 **
**CD8$^+$ T cells**	% of T cells	19.40 (7.34-25.65)	30.10 (24.60-36.10)		4.54	<0.001 ***	28.85 (20.75-35.98)		2.01	0.060	31.00 (24.95-36.35)		4.56	<0.001 ***
	Count /L	0.41 (0.18-0.53)	0.85 (0.56-1.18)	-4.25		<0.001 ***	0.85 (0.59-1.28)		3.83	0.001 **	0.93 (0.55-1.17)	-4.17		<0.001 ***
**Acitvated CD8$^+$ T cells**	% of CD8$^+$ T cells	2.34 (1.03-5.89)	2.64 (1.11-4.49)	-0.06		0.952	2.21 (1.23-6.82)	-0.25		0.805	2.64 (1.10-4.50)	-0.02		0.987
	Count /L	0.01 (0.00-0.02)	0.02 (0.01-0.04)	-2.79		0.005 **	0.03 (0.01-0.04)		1.85	0.115	0.02 (0.01-0.04)	-2.73		0.006 **
**Central memory CD8$^+$ T cell**	% of CD8$^+$ T cells	27.40 (16.48-36.75)	15.60 (8.23-20.80)		-3.79	<0.001 ***	18.75 (3.34-32.23)		-1.31	0.206	15.50 (8.52-20.80)		-4.07	<0.001 ***
	Count /L	0.10 (0.05-0.15)	0.12 (0.08-0.21)	-1.33		0.184	0.19 (0.02-0.31)		1.22	0.272	0.12 (0.08-0.20)	-1.29		0.197
**Effector CD8$^+$ T cell**	% of CD8$^+$ T cells	11.25 (9.38-19.88)	12.00 (8.23-22.60)	-0.08		0.935	10.94 (6.60-31.20)	-0.25		0.805	12.00 (8.28-22.35)	-0.04		0.967
	Count /L	0.05 (0.03-0.09)	0.10 (0.05-0.21)	-2.75		0.006 **	0.12 (0.07-0.18)		2.52	0.021 *	0.10 (0.05-0.23)	-2.65		0.008 **
**Effector memory CD8$^+$ T cell**	% of CD8$^+$ T cells	23.45 (3.69-35.80)	4.06 (2.50-8.01)	-2.95		0.003 **	3.09 (1.08-9.38)		-3.53	0.002 **	4.06 (2.59-8.28)	-2.83		0.005 **
	Count /L	0.06 (0.02-0.14)	0.04 (0.02-0.06)	-0.91		0.363	0.04 (0.01-0.10)		-0.90	0.378	0.04 (0.02-0.06)	-0.78		0.437
**Naive CD8$^+$ T cell**	% of CD8$^+$ T cells	25.15 (15.68-54.90)	54.80 (40.20-65.50)	-2.81		0.005 **	51.60 (29.40-71.65)	-1.81		0.070	55.20 (41.10-65.15)	-2.74		0.006 **
	Count /L	0.10 (0.05-0.23)	0.41 (0.26-0.68)	-4.12		<0.001 ***	0.36 (0.22-0.76)	-2.56		0.011 *	0.42 (0.26-0.66)	-4.05		<0.001 ***
**CD19$^+$ B cells**	% of WBC	6.93 (5.22-9.00)	9.27 (5.45-11.97)		1.07	0.289	9.53 (4.78-13.30)		0.93	0.363	9.00 (5.23-11.84)		1.02	0.310
	Count /L	0.44 (0.34-0.66)	0.77 (0.40-0.98)	-2.31		0.021 *	0.87 (0.49-1.40)		1.95	0.103	0.76 (0.39-0.98)	-2.20		0.028 *
**CD27$^-$ CD38$^+$ B cell**	% of CD19$^+$ B cells	1.25 (0.24-1.73)	2.89 (1.87-4.86)	-3.89		<0.001 ***	1.46 (0.80-3.66)	-1.07		0.284	2.92 (1.88-5.45)	-4.09		<0.001 ***
	Count /L	0.006 (0.001-0.009)	0.019 (0.009-0.041)	-4.10		<0.001 ***	0.013 (0.002-0.055)	-1.40		0.161	0.021 (0.009-0.042)	-4.27		<0.001 ***
**CD27$^+$ CD38$^+$ B cells**	% of CD19$^+$ B cells	0.77 (0.33-1.60)	1.01 (0.27-2.31)	-0.44		0.660	0.30 (0.17-2.82)	-0.78		0.433	1.03 (0.29-2.30)	-0.64		0.519
	Count /L	0.004 (0.001-0.005)	0.006 (0.002-0.018)	-1.18		0.238	0.003 (0.001-0.042)	-0.16		0.869	0.007 (0.002-0.016)	-1.34		0.181
**CD24^{hi} CD38^{hi} Transitional B cell**	% of CD27$^+$ CD38$^+$ B cells	48.70 (37.40-64.00)	5.69 (0.79-25.10)	-4.49		<0.001 ***	6.24 (0.62-13.68)	-3.30		0.001 **	5.69 (0.72-25.20)	-4.31		<0.001 ***
	Count /L	0.00146 (0.00118-0.00250)	0.00030 (0.00004-0.00095)	-3.58		<0.001 ***	00023 (0.00007-0.0015	-2.06		0.039 *	00030 (0.00003-0.0009	-3.55		<0.001 ***
**CD38$^+$ CD20$^-$ Plasmablast**	% of CD27$^+$ CD38$^+$ B cells	42.30 (28.30-53.20)	66.70 (40.00-85.20)	-2.34		0.019 *	77.40 (42.53-85.60)	-1.98		0.048	63.80 (39.60-84.95)	-2.20		0.028 *
	Count /L	0.001 (0.001-0.003)	0.004 (0.001-0.010)	-1.81		0.071	0.002 (0.001-0.036)	-0.66		0.509	0.004 (0.001-0.009)	-1.87		0.062
**CD27$^+$ CD38$^-$ B cells**	% of CD19$^+$ B cells	22.20 (19.10-28.73)	10.30 (7.80-18.10)	-3.91		<0.001 ***	9.98 (7.37-20.88)	-2.31		0.021 *	11.00 (7.39-18.30)	-3.87		<0.001 ***
	Count /L	0.11 (0.07-0.17)	0.08 (0.05-0.12)	-1.95		0.051	0.13 (0.05-0.20)		0.21	0.839	0.07 (0.05-0.12)	-2.26		0.024 *
**IgD$^+$ Memory B cell**	% of CD27$^+$ CD38$^-$ B cells	45.25 (37.28-56.18)	43.20 (22.50-60.30)	-0.40		0.687	57.50 (20.83-65.00)		0.01	0.990	42.40 (20.75-59.60)		-0.93	0.359
	Count /L	0.05 (0.03-0.07)	0.03 (0.01-0.05)	-2.30		0.022 *	0.04 (0.01-0.12)		0.22	0.830	0.03 (0.01-0.05)	-2.41		0.016 *
**IgD$^-$ Memory B cell**	% of CD27$^+$ CD38$^-$ B cells	54.75 (43.83-62.73)	56.80 (39.70-77.50)	-0.40		0.693	42.50 (35.00-79.18)		-0.01	0.990	57.60 (40.40-79.25)		0.92	0.362
	Count /L	0.05 (0.04-0.10)	0.04 (0.02-0.06)	-1.36		0.175	0.06 (0.03-0.10)		0.11	0.917	0.04 (0.02-0.06)	-1.49		0.137
**CD27$^-$ Naive B cell**	% of CD19$^+$ B cells	76.15 (69.58-79.08)	82.80 (73.80-89.30)	-2.68		0.007 **	83.65 (74.63-91.48)		1.94	0.069	82.80 (73.25-88.35)	-2.59		0.010 *
	Count /L	0.32 (0.25-0.48)	0.60 (0.29-0.85)	-2.36		0.018 *	0.77 (0.33-1.11)		2.17	0.075	0.59 (0.29-0.85)	-2.22		0.027 *
**Monocytes**	% of WBC	1.70 (1.42-3.11)	1.28 (0.80-1.88)	-2.70		0.007 **	1.02 (0.56-2.06)	-2.06		0.039 *	1.30 (0.84-1.86)	-2.57		0.010 *
	Count /L	0.13 (0.09-0.23)	0.11 (0.06-0.17)	-1.34		0.180	0.10 (0.07-0.17)		-1.14	0.269	0.11 (0.06-0.17)	-1.32		0.187
**CD16$^+$ Non-classical Monocyte**	% of Monocytes	10.15 (8.17-41.43)	4.94 (2.35-8.31)	-4.05		<0.001 ***	3.33 (1.82-6.22)	-3.18		0.001 **	4.98 (2.59-8.43)	-3.85		<0.001 ***
	Count /L	0.018 (0.007-0.048)	0.005 (0.002-0.009)	-3.78		<0.001 ***	0.003 (0.001-0.005)	-3.38		0.001 **	0.005 (0.003-0.010)	-3.50		<0.001 ***
**CD16$^-$ Classical Monocyte**	% of Monocytes	88.25 (57.58-91.73)	94.00 (90.60-96.60)	-3.73		<0.001 ***	95.60 (93.30-97.63)	-3.05		0.002 **	93.20 (90.55-96.55)	-3.52		<0.001 ***
	Count /L	0.09 (0.07-0.16)	0.10 (0.05-0.16)	-0.19		0.853	0.10 (0.07-0.16)	-0.16		0.869	0.10 (0.05-0.16)	-0.24		0.810
**NK cells**	% of WBC	2.97 (1.19-5.20)	2.23 (1.13-3.62)	-0.97		0.331	1.46 (0.47-2.79)		-1.54	0.141	2.35 (1.24-3.74)	-0.72		0.474
	Count /L	0.20 (0.08-0.31)	0.18 (0.09-0.34)	0.00		1.000	0.11 (0.04-0.36)		-0.42	0.680	0.19 (0.10-0.34)	-0.14		0.891
**CD56^{hi} NK cell**	% of NK cells	10.31 (4.05-93.45)	94.10 (89.20-96.60)	-3.42		0.001 **	87.90 (62.25-94.50)	-1.07		0.283	94.50 (90.10-96.65)	-3.58		<0.001 ***
	Count /L	0.02 (0.00-0.25)	0.17 (0.08-0.33)	-2.79		0.005 **	0.08 (0.02-0.32)	-0.99		0.322	0.18 (0.09-0.33)	-2.90		0.004 **
**CD56^{low} NK cell**	% of NK cells	89.20 (6.22-95.78)	4.63 (1.67-8.86)	-3.95		<0.001 ***	4.40 (1.64-14.18)	-2.56		0.011 *	4.63 (1.67-8.65)	-3.87		<0.001 ***
	Count /L	0.068 (0.020-0.172)	0.006 (0.003-0.016)	-4.56		<0.001 ***	0.005 (0.001-0.022)	-2.89		0.004 **	0.006 (0.003-0.016)	-4.48		<0.001 ***
**DCs**	% of WBC	2.97 (1.19-5.20)	2.23 (1.13-3.62)	-0.97		0.331	1.46 (0.47-2.79)		-1.54	0.141	2.35 (1.24-3.74)	-0.72		0.474
	Count /L	0.20 (0.08-0.31)	0.18 (0.09-0.34)	0.00		1.000	0.11 (0.04-0.36)		-0.42	0.680	0.19 (0.10-0.34)	-0.14		0.891
**CD11c$^+$ Myeloid DC**	% of DCs	54.70 (44.05-68.48)	62.40 (50.00-70.80)	-1.39		0.165	57.25 (25.06-67.88)		-0.39	0.699	62.40 (50.50-72.20)	-1.60		0.109
	Count /L	0.11 (0.05-0.17)	0.10 (0.04-0.19)	-0.22		0.829	0.06 (0.02-0.19)		-0.80	0.436	0.11 (0.05-0.19)	-0.39		0.693
**CD123$^+$ Plasmacytoid DC**	% of DCs	15.35 (8.59-19.50)	17.10 (8.04-23.00)	-0.91		0.363	18.65 (8.15-51.35)		1.18	0.286	17.10 (8.04-22.90)	-0.83		0.408
	Count /L	0.026 (0.006-0.052)	0.034 (0.011-0.069)	-0.54		0.589	0.027 (0.008-0.106)	-0.33		0.741	0.034 (0.011-0.067)	-0.53		0.595
