# Supplementary material for: The immune cell landscape of peripheral blood mononuclear cells from PNS patients
Source: Sci Rep. 2021 Jun 22;11:13083. doi: 10.1038/s41598-021-92573-6 (PMC8219797; doi:10.1038/s41598-021-92573-6)
Supplement: Supplementary file 3 — Supplementary Table 3. [file 41598_2021_92573_MOESM3_ESM.pdf]

Supplemental table 3 Differences in terms of cell subpopulations in patients with SRNS or SSNS before and after treatment

Cell subpopulation	Measure	Group	Before treatment median (IQR)	After treatment median (IQR)	Z value	P value
**WBC**	**Count /L**	**SRNS**	9.94 (8.16-13.59)	12.71 (9.38-14.73)	-1.07 (t Value)	0.294
		**SSNS**	8.70 (6.31-11.16)	10.69 (8.14-12.13)	-1.35	0.176
		**Z Value**	-1.35	0.92 (t Value)	-	-
		**P Value**	0.177	0.365	-	-
**T cells**	**% of WBC**	**SRNS**	35.38 (26.13-40.93)	26.83 (18.25-37.53)	0.90 (t Value)	0.379
		**SSNS**	36.46 (23.85-46.47)	37.49 (18.02-45.17)	0.55 (t Value)	0.582
		**Z Value**	-0.49 (t Value)	-0.98 (t Value)	-	-
		**P Value**	0.626	0.334	-	-
	**Count /L**	**SRNS**	3.45 (1.90-4.46)	2.90 (1.62-4.29)	0.13 (t Value)	0.901
		**SSNS**	2.83 (1.94-3.74)	3.88 (1.53-4.45)	-0.61	0.544
		**Z Value**	-0.67	0.01 (t Value)	-	-
		**P Value**	0.500	0.990	-	-
**CD4$^+$ T cells**	**% of T cells**	**SRNS**	50.20 (42.68-63.13)	47.25 (44.15-56.00)	0.77 (t Value)	0.449
		**SSNS**	57.50 (54.05-66.20)	48.45 (43.60-66.38)	1.72 (t Value)	0.113
		**Z Value**	-1.73 (t Value)	-0.89 (t Value)	-	-
		**P Value**	0.089	0.383	-	-
	**Count /L**	**SRNS**	1.50 (0.90-2.90)	1.50 (0.81-1.98)	0.38 (t Value)	0.707
		**SSNS**	1.64 (1.07-2.37)	1.70 (0.74-2.89)	-0.10	0.920
		**Z Value**	-0.11	-0.49 (t Value)	-	-
		**P Value**	0.914	0.631	-	-
**CXCR3$^+$ CCR6$^-$ TH1 cell**	**% of CD4$^+$ T cells**	**SRNS**	4.02 (1.76-5.54)	3.49 (1.34-7.10)	-0.06	0.951
		**SSNS**	4.31 (2.28-7.20)	8.47 (4.23-9.63)	-1.35	0.176
		**Z Value**	-0.51	-1.14	-	-
		**P Value**	0.608	0.253	-	-
	**Count /L**	**SRNS**	0.07 (0.04-0.08)	0.05 (0.02-0.12)	-0.43	0.670
		**SSNS**	0.07 (0.04-0.12)	0.11 (0.04-0.20)	-0.59	0.558
		**Z Value**	-0.73	-0.84	-	-
		**P Value**	0.466	0.403	-	-
**CXCR3$^-$ CCR6$^-$ TH2 cell**	**% of CD4$^+$ T cells**	**SRNS**	95.55 (94.38-98.28)	92.80 (84.50-98.58)	-0.97	0.330
		**SSNS**	93.80 (89.10-96.00)	92.20 (89.80-95.18)	-0.54	0.592
		**Z Value**	-1.76	-0.40	-	-
		**P Value**	0.079	0.692	-	-
	**Count /L**	**SRNS**	1.42 (0.88-2.82)	1.46 (0.49-1.90)	0.54 (t Value)	0.594
		**SSNS**	1.57 (0.86-2.22)	1.54 (0.39-2.73)	-0.28	0.777
		**Z Value**	-0.11	-0.37 (t Value)	-	-
		**P Value**	0.914	0.718	-	-
**CXCR3$^-$ CCR6$^+$ TH17 cell**	**% of CD4$^+$ T cells**	**SRNS**	0.11 (0.00-0.45)	0.03 (0.00-0.47)	-0.06	0.951
		**SSNS**	0.14 (0.02-0.64)	0.14 (0.06-0.30)	-0.11	0.911
		**Z Value**	-0.95	-1.17	-	-
		**P Value**	0.344	0.242	-	-
	**Count /L**	**SRNS**	0.0014 (0.0000-0.0100)	0.0006 (0.0000-0.0052)	-0.12	0.902
		**SSNS**	0.0017 (0.0003-0.0115)	0.0014 (0.0009-0.0054)	-0.10	0.920
		**Z Value**	-0.76	-0.99	-	-
		**P Value**	0.449	0.320	-	-
**CD25$^+$ CD127^{low} T$_{Reg}$ cells**	**% of CD4$^+$ T cells**	**SRNS**	4.10 (2.28-6.39)	4.97 (3.73-7.35)	-1.00 (t Value)	0.328
		**SSNS**	6.21 (4.10-7.41)	2.77 (2.17-4.36)	-2.73	0.006**
		**Z Value**	-1.41 (t Value)	-2.27	-	-
		**P Value**	0.164	0.023*	-	-
	**Count /L**	**SRNS**	0.05 (0.04-0.14)	0.07 (0.04-0.12)	-0.49	0.626
		**SSNS**	0.08 (0.05-0.15)	0.06 (0.02-0.09)	-2.02	0.043*
		**Z Value**	-1.00	-1.10	-	-
		**P Value**	0.318	0.271	-	-
**CD45RO$^+$ Memory T$_{Reg}$ cell**	**% of CD25$^+$ CD127^{low} T$_{Reg}$ cells**	**SRNS**	19.71 (11.19-25.18)	47.30 (29.70-63.15)	-2.92 (t Value)	0.007**
		**SSNS**	23.50 (14.36-44.70)	37.25 (28.68-44.35)	-0.96	0.337
		**Z Value**	-1.24	1.56 (t Value)	-	-
		**P Value**	0.214	0.131	-	-
	**Count /L**	**SRNS**	0.02 (0.01-0.03)	0.02 (0.01-0.03)	-0.26 (t Value)	0.797
		**SSNS**	0.02 (0.01-0.05)	0.02 (0.01-0.03)	-0.83	0.407
		**Z Value**	-0.84	-0.29	-	-
		**P Value**	0.403	0.775	-	-
**CD45RO$^-$ Naive T$_{Reg}$ cell**	**% of CD25$^+$ CD127^{low} T$_{Reg}$ cells**	**SRNS**	41.05 (29.70-76.03)	54.80 (22.25-67.65)	-0.37	0.715
		**SSNS**	57.90 (41.15-70.85)	61.30 (45.33-91.23)	-1.13	0.258
		**Z Value**	-0.51	-1.28	-	-
		**P Value**	0.608	0.202	-	-
	**Count /L**	**SRNS**	0.02 (0.01-0.11)	0.02 (0.02-0.05)	-0.24	0.808
		**SSNS**	0.05 (0.02-0.08)	0.03 (0.01-0.09)	-0.95	0.342
		**Z Value**	-0.97	-0.26	-	-
		**P Value**	0.331	0.792	-	-
**HLA$^-$DR$^+$ Activated T$_{Reg}$ cell**	**% of CD25$^+$ CD127^{low} T$_{Reg}$ cells**	**SRNS**	5.72 (2.83-57.33)	7.10 (3.32-62.70)	-0.24	0.808
		**SSNS**	7.27 (3.42-12.60)	4.68 (2.23-7.82)	-1.54	0.125
		**Z Value**	-0.24	-1.32	-	-
		**P Value**	0.808	0.187	-	-
	**Count /L**	**SRNS**	0.005 (0.002-0.028)	0.006 (0.002-0.043)	-0.24	0.808
		**SSNS**	0.006 (0.002-0.017)	0.001 (0.001-0.005)	-2.65	0.008**
		**Z Value**	-0.24	-2.16	-	-
		**P Value**	0.808	0.031*	-	-
**Activated CD4$^+$ T cells**	**% of CD4$^+$T cells**	**SRNS**	1.10 (0.48-2.16)	1.12 (0.63-1.37)	-0.12	0.903
		**SSNS**	1.31 (0.83-2.32)	0.89 (0.60-1.28)	-1.54	0.125
		**Z Value**	-0.61	-0.86	-	-
		**P Value**	0.544	0.391	-	-
	**Count /L**	**SRNS**	0.02 (0.01-0.04)	0.01 (0.01-0.02)	-0.67	0.503
		**SSNS**	0.02 (0.01-0.04)	0.01 (0.01-0.02)	-1.86	0.063
		**Z Value**	-0.54	-0.31	-	-
		**P Value**	0.589	0.758	-	-
**Central memory CD4$^+$ T cell**	**% of CD4$^+$T cells**	**SRNS**	21.05 (13.05-28.28)	21.95 (14.48-29.38)	-0.43 (t Value)	0.668
		**SSNS**	23.80 (14.70-30.05)	24.10 (15.51-42.15)	-1.29 (t Value)	0.202
		**Z Value**	-0.24 (t Value)	-0.92 (t Value)	-	-
		**P Value**	0.812	0.368	-	-
	**Count /L**	**SRNS**	0.37 (0.25-0.45)	0.29 (0.17-0.42)	-0.79	0.429
		**SSNS**	0.36 (0.25-0.47)	0.33 (0.26-0.50)	0.24 (t Value)	0.815
		**Z Value**	-0.27 (t Value)	-0.84	-	-
		**P Value**	0.787	0.403	-	-
		**SRNS**	9.79 (8.24-14.50)	12.90 (7.56-33.43)	-0.61	0.543

Effector CD4+ T cell	% of CD4+T cells	SSNS	12.00 (7.11-21.45)	7.52 (5.73-15.48)	-1.66	0.098
		Z Value	-0.39	-1.80	-	-
		P Value	0.695	0.071	-	-
	Count /L	SRNS	0.19 (0.11-0.24)	0.24 (0.08-0.52)	-0.49	0.626
		SSNS	0.19 (0.09-0.52)	0.13 (0.04-0.42)	-1.03	0.303
		Z Value	-0.22	-0.84	-	-
		P Value	0.829	0.403	-	-
Effector memory CD4+ T cell	% of CD4+T cells	SRNS	4.26 (2.28-7.28)	9.67 (3.80-19.13)	-1.58	0.114
		SSNS	4.50 (3.19-7.43)	4.14 (2.71-6.89)	-0.57	0.572
		Z Value	-0.62	-2.07	-	-
		P Value	0.535	0.039*	-	-
	Count /L	SRNS	0.07 (0.04-0.09)	0.12 (0.04-0.25)	-1.28	0.201
		SSNS	0.07 (0.05-0.15)	0.05 (0.04-0.10)	-1.19	0.233
		Z Value	-0.38	-1.76	-	-
		P Value	0.705	0.078	-	-
Naive CD4+ T cell	% of CD4+T cells	SRNS	53.85 (43.50-57.75)	33.00 (20.23-47.60)	-2.31	0.021*
		SSNS	48.40 (41.30-55.80)	46.70 (32.78-51.58)	-0.77	0.443
		Z Value	-1.16	-1.78 (t Value)	-	-
		P Value	0.246	0.086	-	-
	Count /L	SRNS	0.86 (0.39-1.53)	0.41 (0.16-0.69)	-1.70	0.088
		SSNS	0.70 (0.44-1.24)	0.74 (0.25-1.45)	-0.10	0.920
		Z Value	-0.49	-1.23	-	-
		P Value	0.627	0.218	-	-
CD8+ T cells	% of T cells	SRNS	28.85 (20.75-35.98)	40.35 (26.75-48.98)	-1.34 (t Value)	0.194
		SSNS	31.00 (24.95-36.35)	32.75 (26.13-46.35)	-1.57 (t Value)	0.122
		Z Value	-0.67 (t Value)	0.17 (t Value)	-	-
		P Value	0.506	0.866	-	-
	Count /L	SRNS	0.85 (0.59-1.28)	1.11 (0.60-1.77)	-0.87 (t Value)	0.391
		SSNS	0.93 (0.55-1.17)	1.06 (0.55-1.49)	-0.83	0.407
		Z Value	0.00	0.47 (t Value)	-	-
		P Value	1.000	0.643	-	-
Acitvated CD8+ T cells	% of CD8+ T cells	SRNS	2.21 (1.23-6.82)	2.76 (1.49-6.05)	-0.37	0.715
		SSNS	2.64 (1.10-4.50)	3.23 (1.57-5.76)	-0.47	0.635
		Z Value	-0.07	-0.44	-	-
		P Value	0.946	0.660	-	-
	Count /L	SRNS	0.03 (0.01-0.04)	0.02 (0.01-0.09)	-0.18	0.855
		SSNS	0.02 (0.01-0.04)	0.03 (0.01-0.05)	-1.11	0.266
		Z Value	-0.40	-0.22	-	-
		P Value	0.686	0.826	-	-
Central memory CD8+ T cell	% of CD8+ T cells	SRNS	18.75 (3.34-32.23)	13.00 (8.12-20.00)	0.82 (t Value)	0.423
		SSNS	15.50 (8.52-20.80)	10.85 (6.78-22.63)	0.08 (t Value)	0.937
		Z Value	0.84 (t Value)	-0.16 (t Value)	-	-
		P Value	0.406	0.878	-	-
	Count /L	SRNS	0.19 (0.02-0.31)	0.13 (0.05-0.19)	-0.24	0.808
		SSNS	0.12 (0.08-0.20)	0.11 (0.08-0.16)	-0.26	0.793
		Z Value	-0.57	-0.18	-	-
		P Value	0.571	0.860	-	-
Effector CD8+ T cell	% of CD8+ T cells	SRNS	10.94 (6.60-31.20)	18.50 (9.05-43.60)	-1.03	0.301
		SSNS	12.00 (8.28-22.35)	11.65 (9.28-23.63)	-0.16	0.872
		Z Value	-0.46	-0.97	-	-
		P Value	0.646	0.333	-	-
	Count /L	SRNS	0.12 (0.07-0.18)	0.21 (0.06-0.65)	-0.97	0.330
		SSNS	0.10 (0.05-0.23)	0.13 (0.06-0.30)	-0.26	0.793
		Z Value	-0.13	-1.06	-	-
		P Value	0.893	0.291	-	-
Effector memory CD8+ T cell	% of CD8+ T cells	SRNS	3.09 (1.08-9.38)	6.35 (2.21-19.18)	-1.34	0.181
		SSNS	4.06 (2.59-8.28)	2.76 (1.01-4.00)	-2.10	0.036*
		Z Value	-0.85	-2.24	-	-
		P Value	0.395	0.025*	-	-
	Count /L	SRNS	0.04 (0.01-0.10)	0.06 (0.02-0.15)	-0.91	0.361
		SSNS	0.04 (0.02-0.06)	0.02 (0.01-0.03)	-1.72	0.086
		Z Value	-0.54	-1.98	-	-
		P Value	0.589	0.048*	-	-
Naive CD8+ T cell	% of CD8+ T cells	SRNS	51.60 (29.40-71.65)	43.40 (20.55-59.05)	1.18 (t Value)	0.250
		SSNS	55.20 (41.10-65.15)	58.55 (49.90-63.15)	-0.42	0.671
		Z Value	-0.19	-2.00	-	-
		P Value	0.850	0.045*	-	-
	Count /L	SRNS	0.36 (0.22-0.76)	0.30 (0.13-0.77)	-0.43	0.670
		SSNS	0.42 (0.26-0.66)	0.62 (0.29-0.87)	-1.23	0.218
		Z Value	-0.19	-1.28	-	-
		P Value	0.850	0.202	-	-
CD19+ B cells	% of WBC	SRNS	9.53 (4.78-13.30)	7.35 (4.43-10.17)	-0.49	0.626
		SSNS	9.00 (5.23-11.84)	9.04 (4.81-15.58)	-0.70 (t Value)	0.499
		Z Value	0.21 (t Value)	-0.66	-	-
		P Value	0.836	0.509	-	-
	Count /L	SRNS	0.87 (0.49-1.40)	0.83 (0.43-1.40)	-0.06	0.951
		SSNS	0.76 (0.39-0.98)	0.93 (0.44-1.75)	-1.07	0.284
		Z Value	-0.65	-0.13	-	-
		P Value	0.517	0.895	-	-
CD27- CD38+ B cell	% of CD19+ B cells	SRNS	1.46 (0.80-3.66)	0.92 (0.16-3.25)	-0.55	0.584
		SSNS	2.92 (1.88-5.45)	1.56 (0.67-3.81)	-1.90	0.058
		Z Value	-1.65	-0.90	-	-
		P Value	0.100	0.367	-	-
	Count /L	SRNS	0.013 (0.002-0.055)	0.006 (0.001-0.023)	-0.67	0.503
		SSNS	0.021 (0.009-0.042)	0.013 (0.008-0.023)	-1.21	0.225
		Z Value	-0.89	-1.23	-	-
		P Value	0.373	0.218	-	-
CD27+ CD38+ B cells	% of CD19+ B cells	SRNS	0.30 (0.17-2.82)	0.79 (0.25-1.37)	-0.61	0.543
		SSNS	1.03 (0.29-2.30)	0.93 (0.39-1.55)	-0.32	0.746
		Z Value	-0.70	-0.44	-	-
		P Value	0.483	0.660	-	-
	Count /L	SRNS	0.003 (0.001-0.042)	0.003 (0.003-0.011)	-0.43	0.670
		SSNS	0.007 (0.002-0.016)	0.006 (0.004-0.013)	-0.36	0.716
		Z Value	-0.32	-1.19	-	-
		P Value	0.746	0.235	-	-
CD24hi CD38hi Transitional B cell	% of CD27+ CD38+ B cells	SRNS	6.24 (0.62-13.68)	0.58 (0.00-14.98)	-0.97	0.332
		SSNS	5.69 (0.72-25.20)	2.90 (1.63-6.96)	-1.02	0.310
		Z Value	-0.38	-0.56	-	-
		P Value	0.704	0.573	-	-

CD24 CD38 Transitional B cell	Count /L	SRNS	0.00023 (0.00007-0.00156)	0.00005 (0.00000-0.00041)	-1.10	0.273
		SSNS	0.00030 (0.00003-0.00094)	0.00022 (0.00006-0.00052)	-0.67	0.503
		Z Value	-0.04	-1.13	-	-
		P Value	0.968	0.259	-	-
$CD38^{+}$ $CD20^{-}$ Plasmablast	% of $CD27^{+}$ $CD38^{+}$ B cells	SRNS	77.40 (42.53-85.60)	83.50 (59.28-96.25)	-1.10	0.273
		SSNS	63.80 (39.60-84.95)	77.60 (73.55-85.83)	-1.74	0.082
		Z Value	-0.41	-0.68	-	-
		P Value	0.685	0.495	-	-
	Count /L	SRNS	0.002 (0.001-0.036)	0.003 (0.001-0.010)	-0.43	0.670
		SSNS	0.004 (0.001-0.009)	0.005 (0.003-0.010)	-0.95	0.342
		Z Value	-0.20	-1.10	-	-
		P Value	0.840	0.271	-	-
$CD27^{+}$ $CD38^{-}$ B cells	% of $CD19^{+}$ B cells	SRNS	9.98 (7.37-20.88)	23.60 (15.43-42.68)	-2.31	0.021*
		SSNS	11.00 (7.39-18.30)	17.25 (12.33-34.70)	-1.94	0.052
		Z Value	-0.16	1.20 (t Value)	-	-
		P Value	0.871	0.242	-	-
	Count /L	SRNS	0.13 (0.05-0.20)	0.19 (0.08-0.41)	-1.10	0.273
		SSNS	0.07 (0.05-0.12)	0.19 (0.08-0.29)	-2.59	0.010*
		Z Value	-1.13	-0.40	-	-
		P Value	0.257	0.692	-	-
IgD^{+} Memory B cell	% of $CD27^{+}$ $CD38^{-}$ B cells	SRNS	57.50 (20.83-65.00)	34.60 (14.38-56.70)	0.87 (t Value)	0.392
		SSNS	42.40 (20.75-59.60)	65.70 (55.40-71.28)	-2.44	0.015*
		Z Value	0.49 (t Value)	-3.04	-	-
		P Value	0.628	0.002**	-	-
	Count /L	SRNS	0.04 (0.01-0.12)	0.06 (0.02-0.16)	-0.85	0.394
		SSNS	0.03 (0.01-0.05)	0.09 (0.05-0.16)	-3.39	0.001**
		Z Value	-0.73	-1.19	-	-
		P Value	0.466	0.235	-	-
IgD^{-} Memory B cell	% of $CD27^{+}$ $CD38^{-}$ B cells	SRNS	42.50 (35.00-79.18)	65.40 (43.28-85.63)	-0.87 (t Value)	0.394
		SSNS	57.60 (40.40-79.25)	34.30 (28.73-44.60)	-2.41	0.016*
		Z Value	-0.48 (t Value)	-3.04	-	-
		P Value	0.630	0.002**	-	-
	Count /L	SRNS	0.06 (0.03-0.10)	0.10 (0.04-0.19)	-1.34	0.181
		SSNS	0.04 (0.02-0.06)	0.06 (0.03-0.11)	-1.45	0.146
		Z Value	-0.81	-1.23	-	-
		P Value	0.418	0.218	-	-
$CD27^{-}$ Naive B cell	% of $CD19^{+}$ B cells	SRNS	83.65 (74.63-91.48)	70.25 (56.18-77.65)	2.07 (t Value)	0.049
		SSNS	82.80 (73.25-88.35)	79.55 (63.15-85.98)	-1.23	0.218
		Z Value	-0.51	-1.28 (t Value)	-	-
		P Value	0.608	0.211	-	-
	Count /L	SRNS	0.77 (0.33-1.11)	0.61 (0.28-0.86)	-0.85	0.394
		SSNS	0.59 (0.29-0.85)	0.58 (0.34-1.49)	-0.61	0.544
		Z Value	-0.78	-0.44	-	-
		P Value	0.434	0.660	-	-
Monocytes	% of WBC	SRNS	1.02 (0.56-2.06)	2.05 (0.97-3.30)	-1.60 (t Value)	0.123
		SSNS	1.30 (0.84-1.86)	2.92 (1.14-4.84)	-2.16	0.031*
		Z Value	-0.72	-1.39 (t Value)	-	-
		P Value	0.474	0.174	-	-
	Count /L	SRNS	0.10 (0.07-0.17)	0.24 (0.10-0.41)	-2.97 (t Value)	0.007**
		SSNS	0.11 (0.06-0.17)	0.40 (0.10-0.51)	-2.12	0.034*
		Z Value	-0.28	-0.85 (t Value)	-	-
		P Value	0.779	0.403	-	-
$CD16^{+}$ Non-classical Monocyte	% of Monocytes	SRNS	3.33 (1.82-6.22)	4.12 (1.24-9.68)	-0.43	0.670
		SSNS	4.98 (2.59-8.43)	3.10 (1.24-6.33)	-1.30	0.192
		Z Value	-1.32	-0.22	-	-
		P Value	0.186	0.826	-	-
	Count /L	SRNS	0.003 (0.001-0.005)	0.007 (0.003-0.018)	-1.40	0.162
		SSNS	0.005 (0.003-0.010)	0.006 (0.003-0.018)	-0.73	0.463
		Z Value	-1.68	-0.09	-	-
		P Value	0.092	0.930	-	-
$CD16^{-}$ Classical Monocyte	% of Monocytes	SRNS	95.60 (93.30-97.63)	95.35 (88.85-98.50)	-0.33	0.738
		SSNS	93.20 (90.55-96.55)	95.00 (91.38-97.98)	-0.77	0.443
		Z Value	-1.39	-0.26	-	-
		P Value	0.164	0.792	-	-
	Count /L	SRNS	0.10 (0.07-0.16)	0.23 (0.10-0.38)	-2.81 (t Value)	0.010*
		SSNS	0.10 (0.05-0.16)	0.25 (0.09-0.43)	-1.87	0.062
		Z Value	-0.20	-0.25 (t Value)	-	-
		P Value	0.844	0.804	-	-
NK cells	% of WBC	SRNS	1.46 (0.47-2.79)	0.98 (0.52-2.54)	-0.06	0.951
		SSNS	2.35 (1.24-3.74)	1.36 (0.59-2.51)	-1.58	0.113
		Z Value	-1.49	-0.31	-	-
		P Value	0.136	0.758	-	-
	Count /L	SRNS	0.11 (0.04-0.36)	0.13 (0.08-0.33)	-0.11 (t Value)	0.911
		SSNS	0.19 (0.10-0.34)	0.15 (0.07-0.21)	-1.01	0.314
		Z Value	-1.01	-0.13	-	-
		P Value	0.312	0.895	-	-
$CD56^{hi}$ NK cell	% of NK cells	SRNS	87.90 (62.25-94.50)	92.00 (83.20-96.70)	-0.88	0.377
		SSNS	94.50 (90.10-96.65)	96.80 (87.25-98.48)	-1.30	0.192
		Z Value	-1.82	-1.52	-	-
		P Value	0.068	0.129	-	-
	Count /L	SRNS	0.08 (0.02-0.32)	0.11 (0.05-0.27)	0.09 (t Value)	0.929
		SSNS	0.18 (0.09-0.33)	0.14 (0.07-0.20)	-0.90	0.367
		Z Value	-1.17	-0.26	-	-
		P Value	0.244	0.792	-	-
$CD56^{low}$ NK cell	% of NK cells	SRNS	4.40 (1.64-14.18)	3.45 (2.04-8.82)	-0.09	0.927
		SSNS	4.63 (1.67-8.65)	2.47 (1.03-12.36)	-0.96	0.337
		Z Value	-0.11	-0.66	-	-
		P Value	0.914	0.509	-	-
	Count /L	SRNS	0.005 (0.001-0.022)	0.004 (0.001-0.012)	-0.27	0.784
		SSNS	0.006 (0.003-0.016)	0.004 (0.002-0.010)	-1.26	0.208
		Z Value	-0.60	-0.18	-	-
		P Value	0.546	0.860	-	-
DCs	% of WBC	SRNS	1.46 (0.47-2.79)	0.98 (0.52-2.54)	-0.06	0.951
		SSNS	2.35 (1.24-3.74)	1.36 (0.59-2.51)	-1.58	0.113
		Z Value	-1.49	-0.31	-	-
		P Value	0.136	0.758	-	-
	Count /L	SRNS	0.11 (0.04-0.36)	0.13 (0.08-0.33)	-0.11 (t Value)	0.911
		SSNS	0.19 (0.10-0.34)	0.15 (0.07-0.21)	-1.01	0.314
		Z Value	-1.01	-0.13	-	-

		**P Value**	0.312	0.895	-	-
**CD11c⁺ Myeloid DC**	**% of DCs**	**SRNS**	57.25 (25.06-67.88)	60.85 (39.00-78.30)	-0.85 (t Value)	0.403
		**SSNS**	62.40 (50.50-72.20)	59.05 (54.60-76.95)	-0.04	0.968
		**Z Value**	-1.09	-0.84 (t Value)	-	-
		**P Value**	0.274	0.409	-	-
	**Count /L**	**SRNS**	0.06 (0.02-0.19)	0.06 (0.02-0.17)	-0.55	0.584
		**SSNS**	0.11 (0.05-0.19)	0.09 (0.05-0.12)	-0.63	0.529
		**Z Value**	-1.15	-0.57	-	-
		**P Value**	0.250	0.567	-	-
**CD123⁺ Plasmacytoid DC**	**% of DCs**	**SRNS**	18.65 (8.15-51.35)	1.35 (0.02-4.27)	-3.18	0.001**
		**SSNS**	17.10 (8.04-22.90)	5.56 (1.54-15.08)	-2.73	0.006**
		**Z Value**	-0.78	-1.59	-	-
		**P Value**	0.434	0.111	-	-
	**Count /L**	**SRNS**	0.027 (0.008-0.106)	0.001 (0.000-0.009)	-2.50	0.012*
		**SSNS**	0.034 (0.011-0.067)	0.009 (0.001-0.022)	-2.56	0.010*
		**Z Value**	-0.06	-1.20	-	-
		**P Value**	0.955	0.232	-	-
