# Supplementary material for: The immune cell landscape of peripheral blood mononuclear cells from PNS patients
Source: Sci Rep. 2021 Jun 22;11:13083. doi: 10.1038/s41598-021-92573-6 (PMC8219797; doi:10.1038/s41598-021-92573-6)
Supplement: Supplementary file 4 — Supplementary Table 4. [file 41598_2021_92573_MOESM4_ESM.pdf]

Supplemental table 4. the reagents used in immunophenotyping studies

Number of the Product	Product name	Size	Supplier
559925	7-AD Staining Solution	2 mL	BDPharmingen
562444	Hu CD38 BV421 HIT2	100 T	BDPharmingen
564713	Hu CD3 BV510 HT3a	100 T	BDPharmingen
564975	Hu CD4 APC -R700 RPA-T4	100 T	BDPharmingen
557746	Hu CD8 PE -Cy7 RPA-T8	100 T	BDPharmingen
550855	Hu CD45RA APC H1100	100 T	BDPharmingen
565869	Hu CCR7 (CD197) BB515 3D12	100 T	BDPharmingen
555812	Hu HLA-DR PE L243 (G46-6)	100 T	BDPharmingen
562436	Hu CD127 BV421 HIL-7R M21	100 T	BDPharmingen
557864	Hu CCR4 PE-Cy7 1G1	100 ug	BDPharmingen
559865	Hu CD45RO APC UCHL1	100 T	BDPharmingen
564467	Hu CD25 BB515 2A3	100 T	BDPharmingen
550967	Hu CD183 APC 1C6/CXCR3	100 T	BDPharmingen
564479	Hu CD196 (CCR6) BB515 11A9	100 T	BDPharmingen
564977	Hu CD19 APC-R700 HIB19	100 T	BDPharmingen
561646	Hu CD24 PE-Cy7 ML5	50 T	BDPharmingen
559776	Hu CD20 APC 2H7	100 T	BDPharmingen
565243	Hu IgD BB515 IA6-2	100 T	BDPharmingen
555441	Hu CD27 PE M-T271	100 T	BDPharmingen
562517	Hu CD123 BV421 9F5	50 T	BDPharmingen
562947	Hu CD19 BV510 SJ25C1	100 T	BDPharmingen
563067	Hu CD20 BV510 2H7	50 T	BDPharmingen
565139	Hu CD56 APC -R700 NCAM16.2	100 T	BDPharmingen
557744	Hu CD16 PE-Cy7 368	100 T	BDPharmingen
555399	Hu CD14 APC M5E2	100 T	BDPharmingen
564490	Hu CD11c BB515 B Ly6	100 T	BDPharmingen
