# Supplementary figures and images for: The immune cell landscape of peripheral blood mononuclear cells from PNS patients
Source: Sci Rep. 2021 Jun 22;11:13083. doi: 10.1038/s41598-021-92573-6 (PMC8219797; doi:10.1038/s41598-021-92573-6)

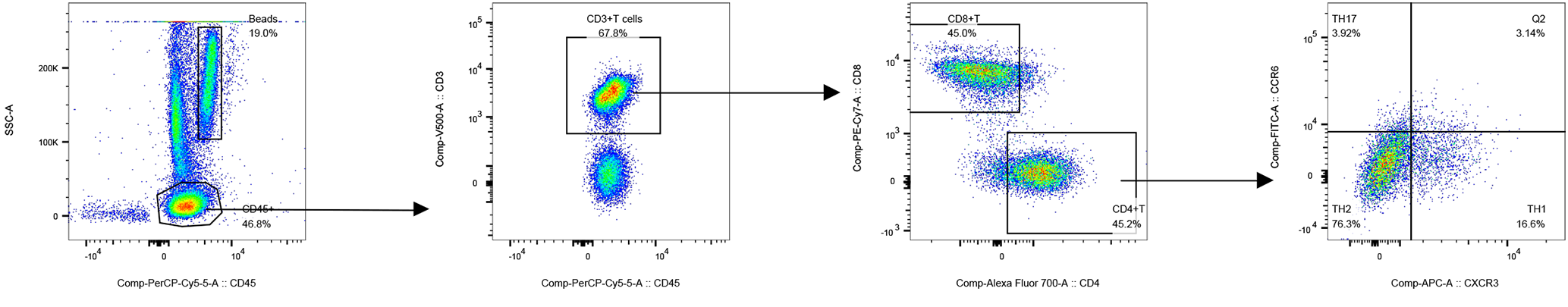

Supplement: Supplementary file 5 — Supplementary Figure 1. [file 41598_2021_92573_MOESM5_ESM.tif]

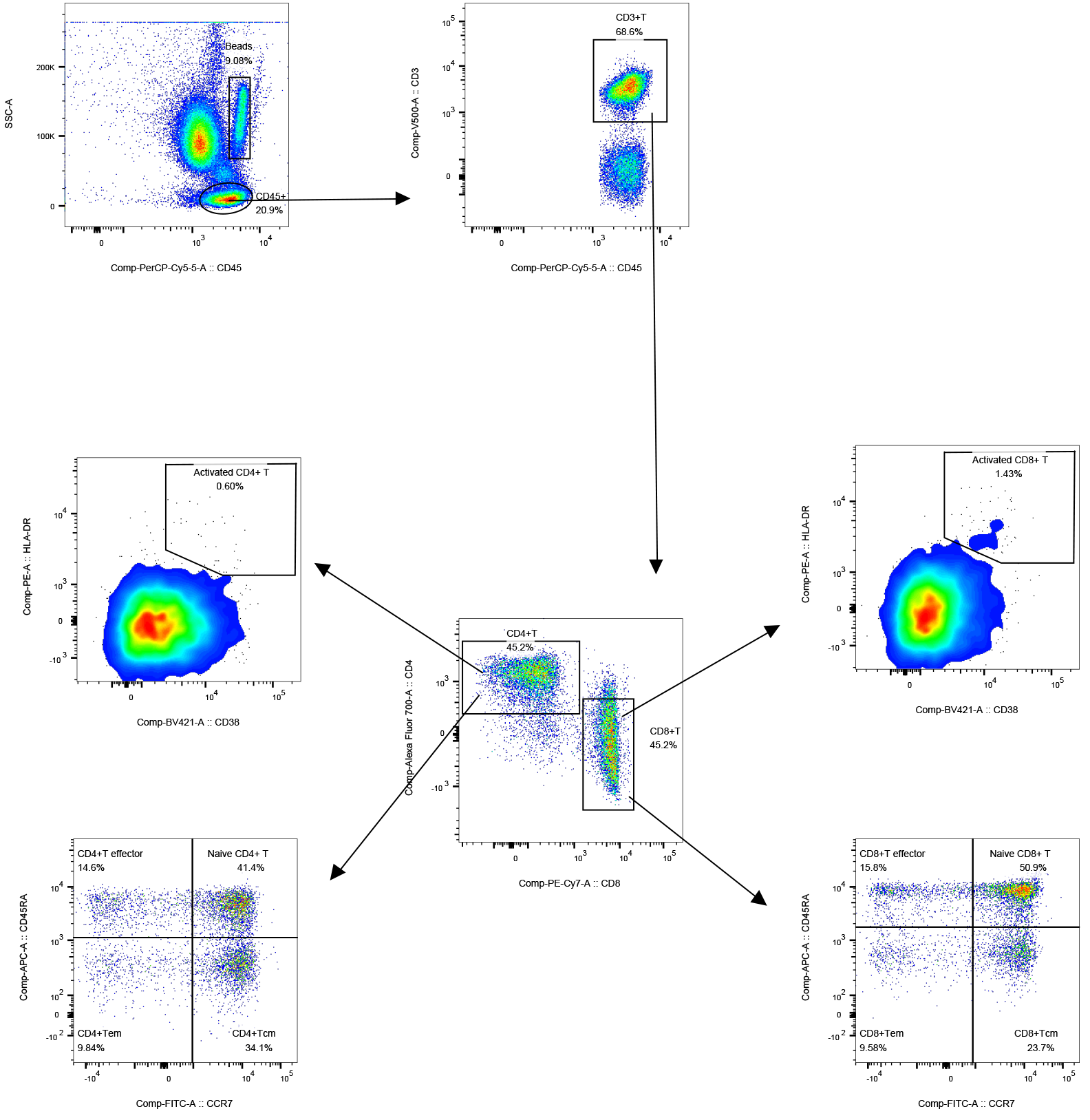

Supplement: Supplementary file 6 — Supplementary Figure 2. [file 41598_2021_92573_MOESM6_ESM.tif]

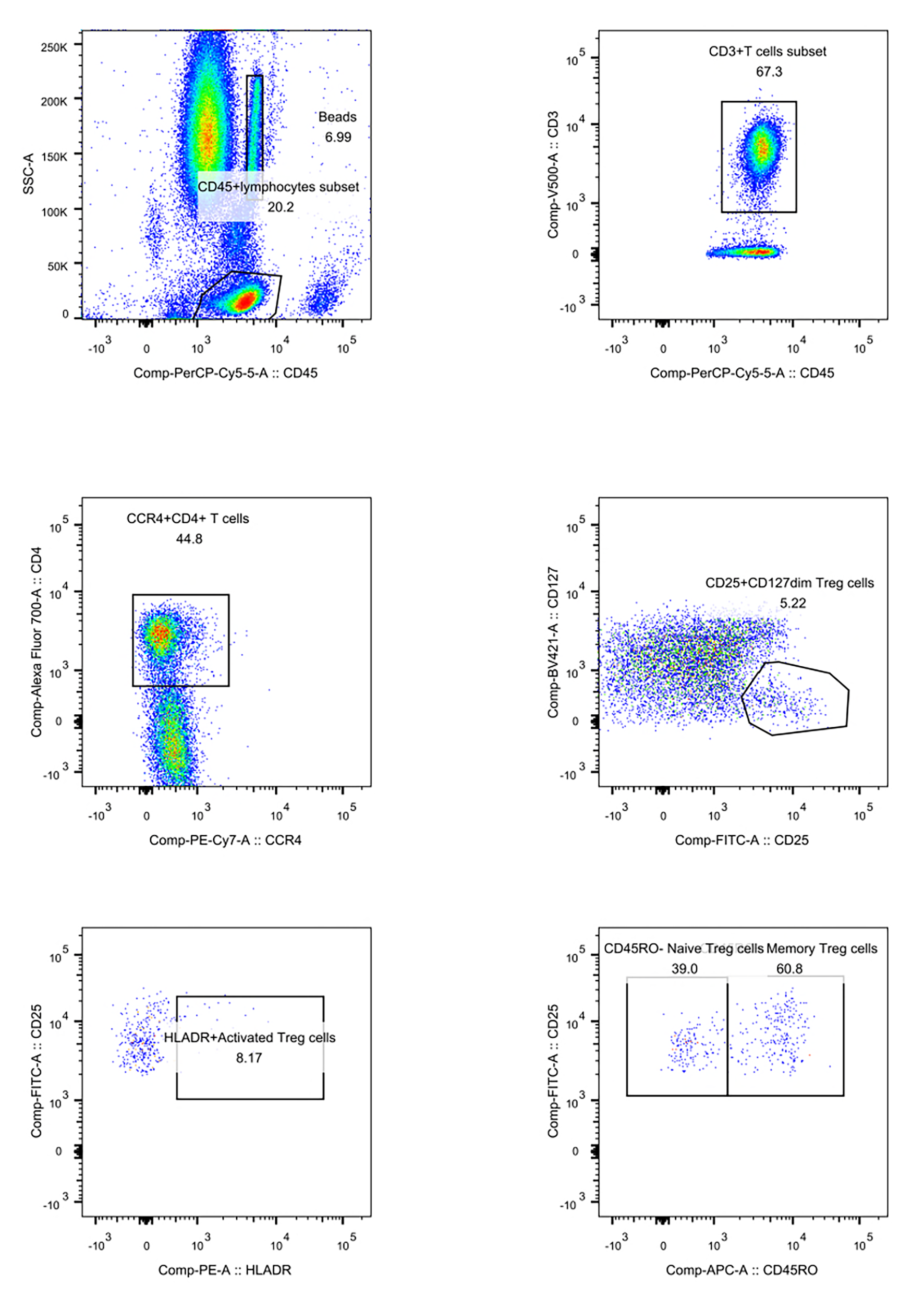

Supplement: Supplementary file 7 — Supplementary Figure 3. [file 41598_2021_92573_MOESM7_ESM.tif]

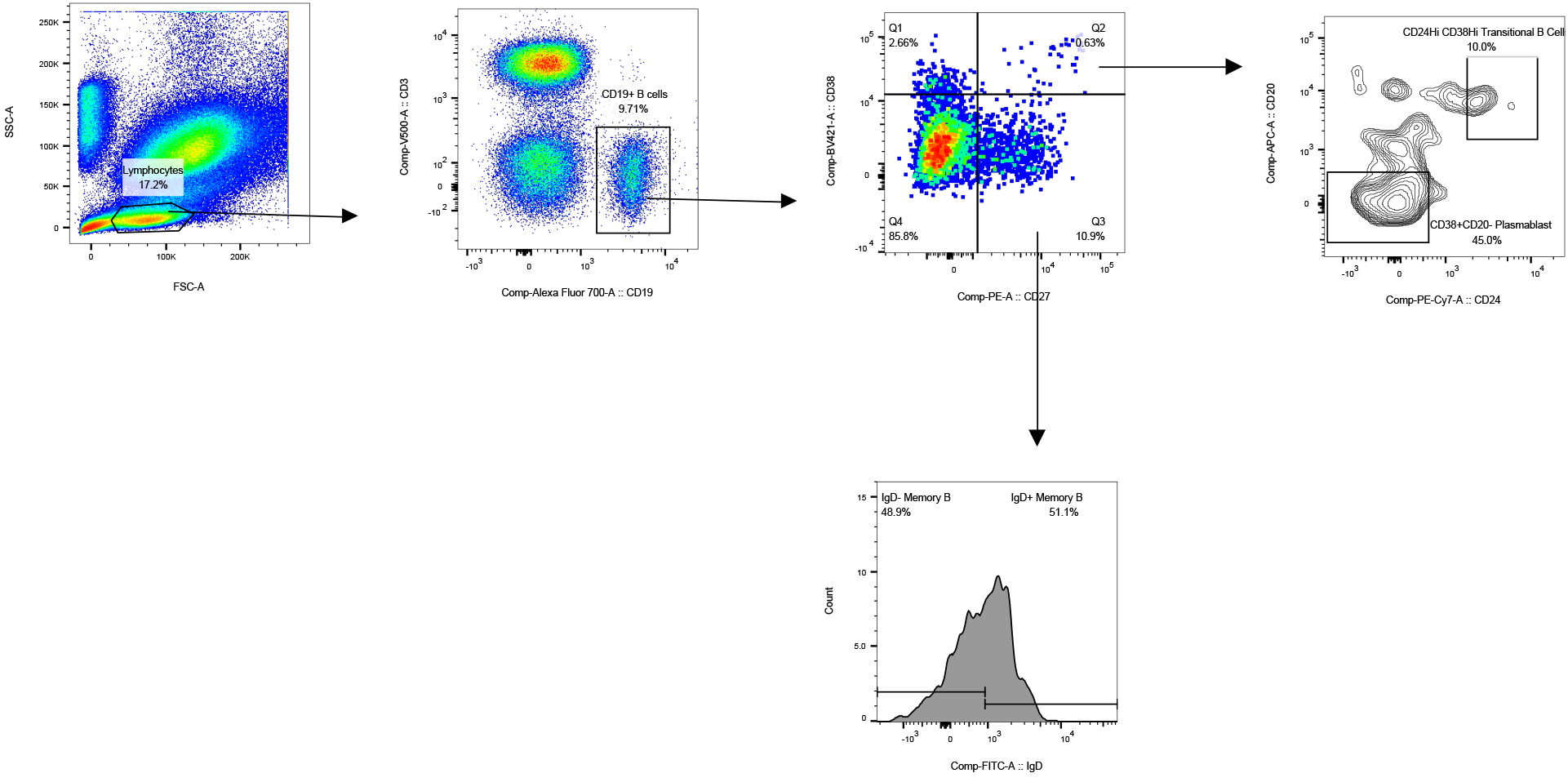

Supplement: Supplementary file 8 — Supplementary Figure 4. [file 41598_2021_92573_MOESM8_ESM.tif]

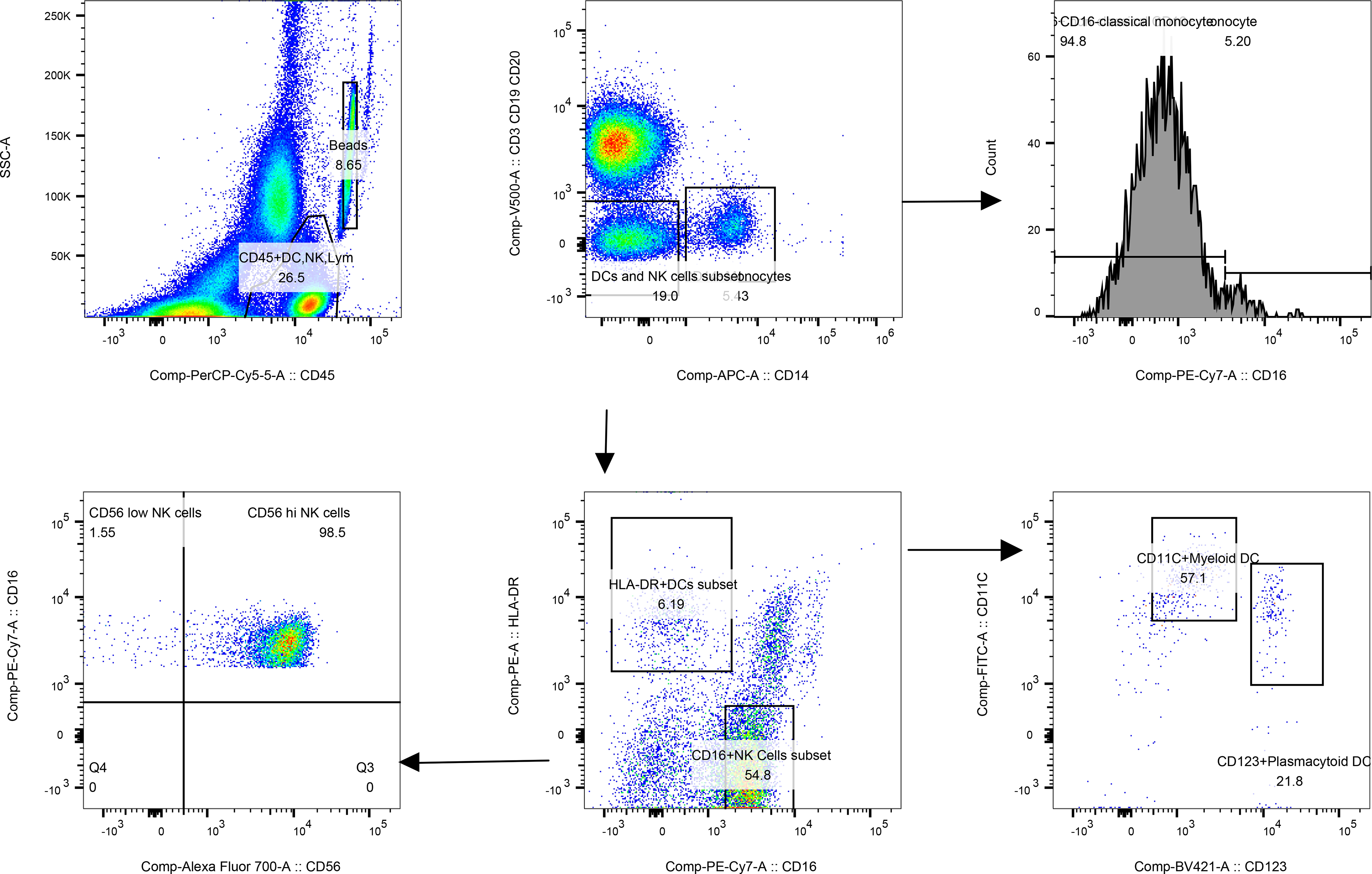

Supplement: Supplementary file 9 — Supplementary Figure 5. [file 41598_2021_92573_MOESM9_ESM.tif]
