# Supplementary material for: The immune cell landscape of peripheral blood mononuclear cells from PNS patients
Source: Sci Rep. 2021 Jun 22;11:13083. doi: 10.1038/s41598-021-92573-6 (PMC8219797; doi:10.1038/s41598-021-92573-6)
Supplement: Supplementary file 10 — Supplementary Legends. [file 41598_2021_92573_MOESM10_ESM.docx]

**Supplemental figure legends**

**Supplemental figure 1. Gating strategy for Th1 cells, Th2 cells, and Th17 cells.**

PBMCs from PNS patients and control subjects were stained with fluorescent anti-CD45, anti-CD3, anti-CD4, anti-CD8, anti-CXCR3, and anti-CCR6. The cells were gated initially on CD45+/SSC^low^ lymphocytes, and then on CD3 + CD4+ T cells, and subsequently on CXCR3-CCR6- TH2 cells and CXCR3+CCR6- TH1 cells.

**Supplemental figure 2. Gating strategy for CD4+T cells and CD8+T cells.**

PBMCs from PNS patients and control subjects were stained with fluorescent anti-CD45, anti-CD3, anti-CD4, anti-CD8, anti-HLADR, CD45RA and anti-CCR7.The cells were gated initially on CD45+/SSC^low^ lymphocytes, and then on CD3 + CD4+ T cells and CD3 + CD8+ T cells. CD4+ T cells were subsequently gated on DR+CD38+ activated CD4+ T cell, CCR7+CD45RA+ naïve CD4+ T cell, CCR7+CD45RA- Central memory CD4+ T cell, CCR7-CD45RA- Effector memory CD4+ T cell and CCR7-CD45RA+ Effector CD4+ T cell. CD8+ T cells were subsequently gated on DR+CD38+ activated CD8+ T cell, CCR7+CD45RA+ naïve CD8+ T cell, CCR7+CD45RA- Central memory CD8+ T cell, CCR7-CD45RA- Effector memory CD8+ T cell and CCR7-CD45RA+ Effector CD8+ T cell.

**Supplemental figure 3. Gating strategy for T_reg_ cells.**

PBMCs from PNS patients and control subjects were stained with fluorescent anti-CD45, anti-CD3, anti-CD4, anti-CCR4, anti-CD25, anti-CD45RO, anti-CD127, and anti-HLA-DR. The cells were gated initially on CD45+/SSC^low^ lymphocytes, and then on CD3+CD4+CCR4+ T and subsequently on CD25+CD127^dim^ T_reg_ cells. The T_reg_ cells were subsequently gated on DR+ activated T_reg_ cells, CD45RO- naïve T_reg_ cells, and CD45RO+ memory T_reg_ cells.

**Supplemental figure 4. Gating strategy for B cells.**

PBMCs from PNS patients and control subjects were stained with fluorescent anti-CD19, anti-CD3, anti-CD38, anti-CD27, anti-CD24, anti-CD20, and anti-IgD. The cells were gated initially on FSC^low^/SSC^low^ lymphocytes, and then on CD19+CD3-B cells, and subsequently on CD27-Naive B cells and CD27+ memory B cells. The memory B cells were gated on IgD+ memory B cells and IgD- memory B cells. The B cells also were gated on CD24^high^CD38^high^ transitional B cells and CD20-CD38+ Plasma blasts.

**Supplemental figure 5. Gating strategy for DC cells, Monocytes, and NK cells.**

PBMCs from PNS patients and control subjects were stained with fluorescent anti-CD19, anti-CD3, anti-CD20, anti-CD14, anti-CD16, anti-CD56, and anti-HLA-DR. The cells were gated initially on CD45+ cells, then on CD14+ Monocytes and CD19-CD3-CD20-CD14- cells (including DCs and NK cells). The Monocytes were gated on CD16- classical monocyte and CD16+ non-classical monocyte. The CD19-CD3-CD20-CD14- cells were gated on HLA-DR+ DCs and CD16+NK cells. The DCs were subsequently gated on CD123+ Plasmacytoid DCs and CD11C+ Myeloid DCs. The CD16+NK cells were subsequently gated on CD56^high^ NK and CD56^low^ NK.
